# Design, Synthesis, and Biological Activity of Novel Chalcone Derivatives Containing an 1,2,4-Oxadiazole Moiety

**DOI:** 10.3389/fchem.2022.943062

**Published:** 2022-07-22

**Authors:** Ling Luo, Dan Liu, Shichao Lan, Xiuhai Gan

**Affiliations:** ^1^ State Key Laboratory Breeding Base of Green Pesticide and Agricultural Bioengineering, Key Laboratory of Green Pesticide and Agricultural Bioengineering, Ministry of Education, Guizhou University, Guiyang, China; ^2^ School of Biological Sciences, Guizhou Education University, Guiyang, China

**Keywords:** plant-parasitic nematodes, chalcone, 1, 2, 4-oxadiazole, nematocidal activity, antiviral activity

## Abstract

To discover a lead compound for agricultural use, 34 novel chalcone derivatives containing an 1,2,4-oxadiazole moiety were designed and synthesized. Their nematocidal activities against *Bursaphelenchus xylophilus*, *Aphelenchoides besseyi*, and *Ditylenchus dipsaci* and their antiviral activities against tobacco mosaic virus (TMV), pepper mild mottle virus (PMMoV), and tomato spotted wilt virus (TSWV) were evaluated. Biological assay results indicate that compounds **A13** and **A14** showed good nematocidal activities against *B. xylophilus*, *A. besseyi*, and *D. dipsaci*, with LC_50_ values of 35.5, 44.7, and 30.2 μg/ml and 31.8, 47.4, and 36.5 μg/ml, respectively, which are better than tioxazafen, fosthiazate, and abamectin. Furthermore, compound **A16** demonstrated excellent protective activity against TMV, PMMoV, and TSWV, with EC_50_ values of 210.4, 156.2, and 178.2 μg/ml, respectively, which are superior to ningnanmycin (242.6, 218.4, and 180.5 μg/ml).

## Introduction

Plant-parasitic nematodes (PPNs) are a very important group of pests that include more than 60 regulated species. These pests are extremely difficult to prevent and cause annual global agricultural losses of roughly $157 billion (Abad, et al., 2008; Bernard, et al., 2017; Abd-Elgawad, 2020; Kantor, et al., 2022). At present, the application of chemical nematicides is the most reliable and effective method to control PPNs. However, these treatments are mainly based on highly toxic organophosphorus and carbamate nematocides, such as fosthiazate, cadusafos, fenamiphos, dazomet, aldicarb, oxamyl, and so on. The long-term use, overuse, and misuse of these nematicides have not only led to poor control effect and serious resistance but have also seriously harmed the environment (Ntalli and Caboni, 2012; Chen, et al., 2020). Meanwhile, plant virus disease, as a “plant cancer,” can lead to considerable crop loss (Chen, et al., 2016). Although Ribavirin is widely used to prevent plant virus disease, its inhibitory effect to a virus is less than 50% at 500 mg/L (Wang, et al., 2012). To date, we still lack effective and low toxicity nematicides and antiviral agents for use in agricultural production. In addition, a combined infection of PPNs and viruses will significantly increase the loss of agricultural production. Hence, the discovery of new, environmentally friendly, and efficient nematicides and antiviral agents is key to controlling plant nematode and virus diseases.

Natural products have often been the source of new drug discovery and have the benefits of low toxicity, easy decomposition, and are environmentally friendly (Leonard and Stephen, 2007; Qian, et al., 2010; Chen, et al., 2020). As one of the most important natural products, chalcone is widely found in plants (Du, et al., 2013) and has various biological activities, including anticancer (Kim, et al., 2013; Wang, et al., 2015), antibacterial (Wei, et al., 2016), antifungal (Lahtchev, et al., 2008) and antiviral (Park, et al., 2011) effects in medicine. In addition, chalcone and its derivatives have insecticidal (Thirunarayanan, et al., 2010), nematicidal (Attar, et al., 2011; Nunes, et al., 2013; Caboni, et al., 2016), antiviral (Du, et al., 2013), and other agricultural activities. In our previous work, we reported that chalcone derivatives containing 1,3,4-oxadiazole/thiadiazole, purine, and ferulic acid moieties have excellent antiviral activities (Gan, et al., 2017a; Gan, et al., 2017b).

As an important heterocyclic compound, 1,2,4-oxadiazole has been widely studied by pesticide scientists with a wide range of biological activities, such as herbicidal (Hang, et al., 2014), antibacterial (Karad, et al., 2017), antifungal (Yang, et al., 2021), and insecticidal activities (Fernandes, et al., 2020), among others. Tioxazafen, which is one of the 1,2,4-oxadiazole compounds, was designed by Monsanto as a new type of seed treatment agent to control nematodes in soybean, corn, and cotton (Slomczynska, et al., 2015). However, Tioxazafen has not formally been used on a large scale in agricultural production. To enhance the flexibility of the structure of Tioxazafen and discover the high activity 1,2,4-oxadiazole compound, some 1,2,4-oxadiazole derivatives containing 1,3,4-oxadiazole/thiadiazole and amide moieties with good nematocidal, antibacterial, and antifungal activities have been synthesized (Zhu, et al., 2020; Liu, et al., 2022).

**FIGURE 1:**
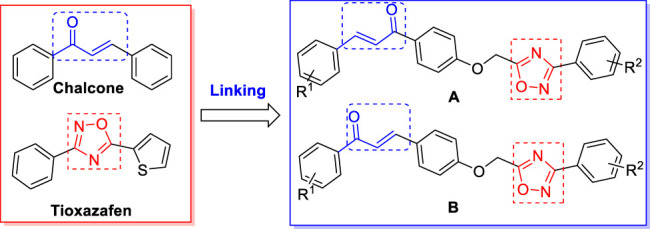
Design of the target compounds.

Based on the biological activity of chalcone and 1,2,4-oxadiazole derivatives, the current study aims to further improve the nematocidal activity, and to extend the biological activity of chalcone and 1,2,4-oxadiazole moieties. In particular, a 1,2,4-oxadiazole fragment is introduced to the chalcone skeleton to obtain 34 novel chalcone derivatives containing 1,2,4-oxadiazole moiety (Figure 1). Their nematocidal activities against *Bursaphelenchus xylophilu*s, *Aphelenchoides besseyi,* and *Ditylenchus dipsaci* and their antiviral activities to tobacco mosaic virus (TMV), pepper mild mottle virus (PMMoV), and tomato spotted wilt virus (TSWV) were then evaluated.

## Materials and Methods

### General Information

The melting points of the compounds were determined on an X-4B microscope melting point apparatus and were uncorrected (Shanghai Electrophysics Optical Instrument Co., Ltd., Shanghai, China). The ^1^H NMR and ^13^C NMR spectra data of the compounds were recorded on a Bruker DPX-400 spectrometer (Bruker, Billerica, MA, United States), using DMSO-*d*_6_ as solvents and tetramethylsilane as an internal standard. The high-resolution mass spectrometer (HRMS) data of the compounds were obtained with a Thermo Scientific Q-Exactive (Thermo Scientific, Missouri, MOThermo, United States). Reactions were detected by thin-layer chromatography (TLC) and visualized under UV light at 254 nm. Chromatography was conducted on silica gel 200–300 mesh.

### Synthesis

#### Preparation Procedure for Intermediates **2** and **6**

**SCHEME 1:**
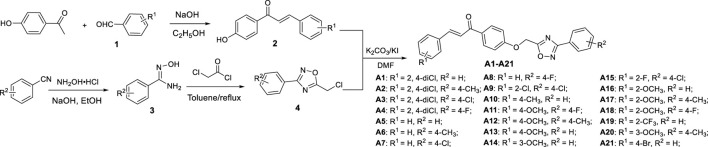
Synthetic process and the experimental method of the target compounds **A1**−**A21**.

**SCHEME 2:**
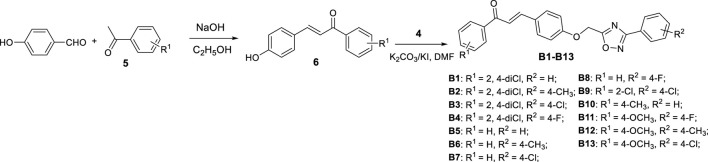
Synthetic process and the experimental method of the target compounds **B1**−**B13**.

As shown in Schemes 1 and 2, the chalcone intermediates **2** and **6** were obtained according to our previously reported methods (Gan, et al., 2017c). First, 0.2 M aqueous sodium hydroxide solution (22 mmol) was added to a solution of 4-hydroxyacetophenone or 4-hydroxybenzaldehyde (20 mmol) and various substituted benzaldehyde or acetophenone (20 mmol) in 20 ml ethanol, and then stirred at room temperature for 12 h. Second, upon reaction completion (monitored by TLC), the mixture was poured into ice-water and acidified to a pH value of 2‒3 by dropwise addition of aqueous HCl, filtered, washed, and dried to obtain intermediates **2** and **6**.

#### Preparation Procedure for Intermediate **4**

An aqueous solution of sodium hydroxide (50 mmol) was added to a solution of hydroxylamine hydrochloride (50 mmol) in 30 ml ethanol and stirred at room temperature. Various benzonitriles (50 mmol) were then added to the mixture. The mixture was heated to reflux and monitored with TLC. After completion of the reaction, the precipitated product was filtrated and the filtrate was concentrated under reduced pressure. The residue was dissolved with toluene and chloroacetyl chloride (50 mmol) was added dropwise into the mixture in an ice bath, and then refluxed for 10 h. The solvent was removed and the residue was dissolved with dichloromethane and washed with brine. The organic layer was then dried and further purified by column chromatography to afford intermediate **4**.

#### Preparation Procedure for the Target Compounds **A1−A21** and **B1−B13**

A solution of intermediate **4** (2.0 mmol) in 5 ml DMF was added to a solution of intermediate **2** or **6** (2.0 mmol), K_2_CO_3_ (2.2 mmol) in 5 ml *N*,*N*-dimethylformamide (DMF) and warmed to 40°C for 4 h. Upon completion of reaction, the mixture was poured into ice-water, filtered, and recrystallized from methanol to give the pure target compounds **A1−A21** and **B1−B13**. The physical properties, ^1^HNMR, ^13^CNMR, and HRMS for title compounds are reported in the Supplementary Data. The spectral data of **A1** and **B1** are shown below.

(*E*)-3-(2,4-dichlorophenyl)-1-(4-((3-phenyl-1,2,4-oxadiazol-5-yl)methoxy)phenyl)prop-2-en-1-one (**A1**). White powder; m.p. 154–155°C; yield 89%; ^1^H NMR (400 MHz, DMSO-*d*_6_): *δ* 8.02 (d, *J* = 6.4 Hz, 2H), 7.79 (d, *J* = 8.8 Hz, 2H), 7.77 (d, *J* = 1.6 Hz, 1H), 7.59–7.58 (m, 5H), 7.40 (d, *J* = 16.0 Hz, 1H), 7.17 (d, *J* = 9.2 Hz, 2H), 7.16 (d, *J* = 15.6 Hz, 1H), 5.70 (s, 2H); ^13^C NMR (101 MHz, DMSO-*d*_*6*_): *δ* 192.83, 175.99, 168.85, 160.07, 147.02, 138.07, 135.90, 132.31, 131.63, 131.50, 131.50, 131.10, 130.12, 129.84, 129.84, 128.38, 128.10, 127.56, 127.56, 126.20, 124.95, 115.80, 115.80, 61.39. HRMS (ESI) m/z for C_24_H_17_O_3_N_2_Cl_2_ [M+H]^+^ calcd: 451.06107, found: 451.05978.

(*E*)-1-(2,4-dichlorophenyl)-3-(4-((3-phenyl-1,2,4-oxadiazol-5-yl)methoxy)phenyl)prop-2-en-1-one (**B1**). Faint yellow powder; m.p. 126–127°C; yield 56%; ^1^H NMR (400 MHz, DMSO-*d*_6_): *δ* 8.33–8.27 (m, 3H), 8.12–7.98 (m, 4H), 7.79 (d, *J* = 1.6 Hz, 1H), 7.67–7.59 (m, 4H), 7.33 (d, *J* = 8.8 Hz, 2H), 5.82 (s, 2H); ^13^C NMR (101 MHz, DMSO-*d*_*6*_): *δ* 187.54, 175.87, 168.27, 161.79, 137.19, 135.98, 135.57, 132.31, 131.92, 131.67, 131.67, 131.65, 130.30, 129.95, 129.84, 129.84, 128.39, 127.57, 127.57, 126.20, 125.72, 115.39, 115.39, 61.51. HRMS (ESI) m/z for C_24_H_17_O_3_N_2_Cl_2_ [M+H]^+^ calcd: 451.06107, found: 451.05972.

### Nematocidal Activity Test

*B. xylophilus*, *A. besseyi,* and *D. dipsaci* were bred with potato dextrose agar–Botrytis cinerea provided from the Fine Chemical Research and Development Center of Guizhou University (Guizhou, China). The nematocidal bioassays of these target compounds was tested based on the previous reported methods with minor modification (Wei, et al., 2021). The compound was dissolved with 50 μl DMF, and was then diluted with 1% Tween-80 to obtain 50 and 10 μg/ml concentrations. Meanwhile, fosthiazate and tioxazafen were used as positive controls at the same concentrations and without compounds solution as a negative control group. Then, 10 µl of nematode suspension with 50 nematodes and 300 µl of the solution were added to the corresponding hole of 48-well plates, each treatment was repeated three times, and they were then placed in a biochemical incubator at 27°C for dark light culture. After 48 h, the dead nematodes were counted and the corrected mortality was calculated with the following formula:

Corrected mortality % = [(mortality of treatment % − mortality of negative control %)/ (1 − mortality of negative control %)] × 100

### Antiviral Activity Test

*Nicotiana tabacum* cv. K326, *Nicotiana benthamiana*, and *Nicotiana glutinosa* L. plants were cultivated in a greenhouse. *N. tabacum* cv. K326 was used to determine systemic TMV infection, and *N. benthamiana* was used to determine systemic PMMoV and TSWV infection. *N. glutinosa* L. was used as a local lesion host to evaluate the antiviral activity against TMV, PMMoV, and TSWV when the plants grew to 5–6 leaf stages. TMV, PMMoV, and TSWV were purified by the Gooding method (Gooding and Hebert, 1967) and the curative, protective activities of compounds were performed with the reported methods at 500 μg/ml (Song, et al., 2005; Zan, et al., 2021; Shi, et al., 2022). The EC_50_ values of the antiviral activity at concentrations of 500, 250, 125, 62.5, and 31.25 μg/ml were then calculated. The positive controls included ribavirin and ningnanmycin. Measurements were performed in triplicates.

## Results and Discussion

### Chemistry

The influence of the catalyst, temperature, and solvent for preparation compound **A1** was tested and evaluated to obtain the facile, high efficiency, and yield synthetic method of the target compound; the results are given in Table 1. The results indicate that the yield of compound **A1** was affected by the catalyst, solvent, and temperature. The optimum synthesis condition is catalyst as K_2_CO_3_/KI, DMF as solvent, and reaction for 6 h at 80°C. Under this condition, the yield of compound **A1** achieved 89%. The other compounds were then prepared with the same condition. The structures of all of the compounds were identified with ^1^H NMR, ^13^C NMR, and HRMS. Two doublets appear in the ^1^H NMR data of compound **A1**, 7.40 (*J* = 16.0 Hz) ppm and 7.16 (*J* = 15.6 Hz) ppm, which indicate the presence of the HC=CH group. The proton of CH_2_ appears as a singlet at 5.70 ppm. Meanwhile, the 192.83 ppm peak of the ^13^C NMR data indicates the presence of the C=O group, and the 170.32 and 167.66 ppm peaks indicate the presence of C proton in the 1,2,4-oxadiazol group. The 61.39 ppm peak indicates the presence of the C proton of the CH_2_ group. Furthermore, compound **A1** was confirmed correctly with HRMS data of the [M+H]^+^ as 451.05978, the calculated value was 451.06107.

**TABLE 1:** The reaction conditions for compound **A1** were optimized.

Entry	Catalyst	Solvent	Temperature/°C	Yield^a^ (%)
1	K_2_CO_3_	CH_3_CN	r.t	32
2	Na_2_CO_3_	CH_3_CN	r.t	15
3	NaOH	CH_3_CN	r.t	21
3	K_2_CO_3_/KI	CH_3_CN	r.t	38
4	K_2_CO_3_/KI	CH_3_CN	80	71
5	K_2_CO_3_	DMF	r.t	39
6	K_2_CO_3_/KI	DMF	r.t	56
7	K_2_CO_3_	DMF	60	85
8	K_2_CO_3_	(CH_3_)_2_CO	56	48
9	K_2_CO_3_	DMF	80	83
10	K_2_CO_3_/KI	DMF	60	89

a Isolated yield.

### Nematocidal Activity Test

The results of nematocidal activities of compounds are given in Table 2. As shown in Table 2, compounds **A13**, **A14**, and **B3** exhibited higher nematocidal activity against *B. xylophilus* at 50 μg/ml, the corrected mortalities were 100%, 100%, and 51.8%, respectively, which are superior to those of tioxazafen (34.3%), fosthiazate (43.9%), and abamectin (49.4%). Meanwhile, compounds **A13**, **A14**, **B6**, and **B12** showed good nematocidal activity against *A. besseyi* at 50 μg/ml, with corrected mortalities of 100%, 100%, 70.8%, and 59.6%, which are better than tioxazafen (40.0%) and abamectin (42.3%). In addition, compounds **A13**, **A14**, and **B11** possessed desired nematocidal activity against *D. dipsaci*, with corrected mortalities of 100%, 100%, and 41.0%, respectively, which are superior to tioxazafen (29.0%), fosthiazate (33.3%), and abamectin (33.6%). However, there was dissatisfactory nematocidal activity of all compounds against *B. xylophilus*, *A. besseyi*, and *D. dipsaci* at 10 μg/ml.

**TABLE 2:** Nematicidal activity of compounds **A1**−**A21** and **B1**−**B13**.^a^

Compd.	Corrected mortality ±SD (%)^b^					
	*B. xylophilus*		*A. besseyi*		*D. dipsaci*	
	50 μg/ml	10 μg/ml	50 μg/ml	10 μg/ml	50 μg/ml	10 μg/ml
**A1**	26.6 ± 3.8	—	32.0 ± 6.5	—	—	—
**A2**	—	—	—	—	22.4 ± 2.9	—
**A3**	—	—	—	—	—	—
**A4**	37.7 ± 3.9	—	—	—	—	—
**A5**	38.8 ± 4.9	—	32.0 ± 6.5	—	23.1 ± 1.3	—
**A6**	30.6 ± 5.8	—	21.2 ± 2.4	—	20.6 ± 5.2	—
**A7**	—	—	24.6 ± 5.3	20.5 ± 2.2	24.7 ± 0.4	—
**A8**	—	—	21.5 ± 2.3	—	22.6 ± 8.0	—
**A9**	48.9 ± 7.4	26.9 ± 5.7	—	—	30.7 ± 3.5	24.2 ± 5.9
**A10**	26.9 ± 5.2	—	—	—	30.7 ± 5.3	—
**A11**	20.7 ± 4.7	—	24.1 ± 8.6	—	22.0 ± 2.8	—
**A12**	41.0 ± 6.9	23.5 ± 4.1	—	—	27.7 ± 8.2	—
**A13**	100	25.8 ± 4.9	100	25.8 ± 5.9	100	25.8 ± 1.9
**A14**	100	25.1 ± 5.6	100		100	24.1 ± 1.6
**A15**	47.9 ± 5.7	32.1 ± 7.7	—	—	—	—
**A16**	21.5 ± 1.5	—	—	—	—	—
**A17**	—	—	—	—	—	—
**A18**	36.5 ± 9.5	25.4 ± 1.0	—	—	25.4 ± 1.0	—
**A19**	44.9 ± 6.1	23.3 ± 6.7	—	—	—	—
**A20**	25.5 ± 5.5	—	—	—	—	—
**A21**	23.0 ± 4.5	—	—	—	23.4 ± 6.2	—
**B1**	29.9 ± 6.5	—	25.2 ± 7.1	—	22.4 ± 6.0	—
**B2**	37.7 ± 6.7	—	21.8 ± 3.3	—	28.8 ± 1.2	—
**B3**	51.8 ± 5.8	—	25.6 ± 4.0	—	25.5 ± 3.8	—
**B4**	28.1 ± 7.8	—	25.0 ± 3.3	—	24.9 ± 9.0	—
**B5**	31.1 ± 5.8	—	29.5 ± 2.8	20.8 ± 7.3	33.2 ± 1.5	23.3 ± 2.7
**B6**	37.0 ± 1.1	—	70.8 ± 1.8	—	29.9 ± 6.2	20.7 ± 6.6
**B7**	27.8 ± 6.3	—	—	—	21.6 ± 4.4	—
**B8**	—	—	—	—	25.3 ± 5.9	—
**B9**	—	—	—	—	29.3 ± 3.4	—
**B10**	—	—	22.9 ± 2.8	—	22.4 ± 4.7	—
**B11**	—	—	31.5 ± 4.3	—	41.0 ± 7.4	22.2 ± 4.1
**B12**	—	—	59.6 ± 9.2	—	—	—
**B13**	25.1 ± 1.1	—	35.8 ± 1.7	—	33.0 ± 6.5	—
Tioxazafen^b^	34.3 ± 7.7	—	40.0 ± 6.1	20.1 ± 2.5	29.0 ± 3.7	—
Fosthiazate^b^	43.9 ± 5.2	23.2 ± 9.8	—	—	33.3 ± 1.6	—
Abamectin^b^	49.4 ± 6.3	31.9 ± 4.2	42.3 ± 2.0	22.2 ± 3.2	33.6 ± 1.3	20.2 ± 3.3

a Average of three replicates.

b The commercial antiviral agents tioxazafen, fosthiazate, and abamectin were used for comparison of activity.

“—” No activity or corrected mortality <20%.

To further confirm their nematicidal activities of compounds **A13** and **A14**, the LC_50_ values of compounds **A13** and **A14** against *B. xylophilus*, *A. besseyi*, and *D. dipsaci* were evaluated as tioxazafen, fosthiazate, and abamectin for positive controls; the results are given in Table 3. As shown in Table 3, compounds **A13** and **A14** had LC_50_ values of 35.5, 44.7, and 30.2 *μ*g/ml and 31.8, 47.4, and 36.5 *μ*g/ml against *B. xylophilus*, *A. besseyi*, and *D. dipsaci*, respectively, which are superior to tioxazafen, fosthiazate, and abamectin. In addition, the results indicate that **A** series compounds have better nematocidal activity than the **B** series compound. However, there is no obvious regularity between activity and structure.

**TABLE 3:** The LC_50_ values of nematicidal activity of compounds.

Compd.	LC_50_ (μg/ml)^a^		
	*B. xylophilus*	*A. besseyi*	*B. cinerea*
**A13**	35.5 ± 3.5	44.7 ± 5.4	30.2 ± 2.0
**A14**	31.8 ± 0.9	47.4 ± 2.5	36.5 ± 0.7
Tioxazafen^b^	>200	>200	>200
Fosthiazate^b^	>200	>200	>200
Abamectin^b^	103.8 ± 1.5	>200	106.2 ± 2.1

a Average of three replicates.

b The commercial antiviral agents tioxazafen, fosthiazate, and abamectin were used for comparison of activity.

### Antiviral Activity Test

The antiviral activities of the target compounds were performed with the half leaf blight spot method and the results are given in Tables 4 and 5. As shown in Table 4, compounds **A4**, **A11**, **A16**, **A18**, and **A20** exhibited better curative activity against TMV at 500 μg/ml, with values of 49.8%, 53.6%, 57.2%, 52.3%, and 51.2%, respectively, which are superior than those of ribavirin (39.9%) and ningnanmycin (49.8%). These compounds also showed good protective activity to TMV, the inhibitory was 64.5%, 67.9%, 68.6%, 65.2%, and 67.1%, respectively, which are better than those of ribavirin (51.2%) and ningnanmycin (61.3%). Meanwhile, compounds **A4**, **A11**, **A16**, **A18**, and **B11** showed desirable curative action against PMMoV, the values were 52.3%, 53.6%, 56.5%, 55.6%, and 52.9%, which are better than those of ribavirin (31.6%) and ningnanmycin (51.8%). Furthermore, compounds **A4**, **A11**, **A16**, **A18**, **A20,** and **B11** showed excellent protective activity against PMMoV, with values of 67.1%, 65.6%, 71.8%, 70.2%, 68.1%, and 63.7%, respectively, which are superior to ribavirin (48.8%) and ningnanmycin (63.3%). Unfortunately, the curative effect of compounds to TSWV was dissatisfactory. However, compounds **A16** (69.5%) and **A18** (65.6%) showed better protective activity against TSWV than ribavirin (46.2%) and ningnanmycin (65.1%). The results of the EC_50_ values (Table 5) indicate that compounds **A16** and **A18** showed excellent curative and protective activities against TMV and PMMoV, with EC_50_ values of 368.7 and 210.4 μg/ml, 310.8 and 156.2 μg/ml, 410.5 and 251.2 μg/ml, and 345.6 and178.2 μg/ml, respectively, which are superior to ningnanmycin (420.5 and 242.6 μg/ml, and 415.8 and 218.4 μg/ml, respectively). In addition, compound **A16** (178.9 μg/ml) showed better protective activity against TSWV than ningnanmycin (180.5 μg/ml).

**TABLE 4:** Antiviral activities of compounds **A1**−**A21**and **B1**−**B13** at 500 μg/ml.^a^

Compd.	TMV		PMMoV		TSWV	
	Curative activity (%)	Protective activity (%)	Curative activity (%)	Protective activity (%)	Curative activity (%)	Protective activity (%)
**A1**	45.6 ± 1.9	60.3 ± 2.5	39.5 ± 1.1	56.1 ± 1.8	27.8 ± 3.0	46.5 ± 2.2
**A2**	38.9 ± 2.9	49.8 ± 1.1	45.3 ± 2.5	57.2 ± 1.4	35.7 ± 1.0	45.6 ± 2.3
**A3**	36.1 ± 2.3	47.2 ± 2.6	40.6 ± 1.7	49.3 ± 1.8	32.9 ± 2.7	48.0 ± 1.9
**A4**	49.8 ± 1.1	64.5 ± 3.4	52.3 ± 2.5	67.1 ± 2.3	46.7 ± 1.9	63.1 ± 2.8
**A5**	23.6 ± 2.6	54.2 ± 1.9	39.8 ± 1.9	60.2 ± 2.2	31.2 ± 1.3	54.8 ± 2.9
**A6**	37.8 ± 2.1	54.1 ± 2.9	43.8 ± 3.1	59.2 ± 3.1	33.3 ± 1.7	51.2 ± 2.5
**A7**	30.6 ± 1.8	49.5 ± 2.5	36.3 ± 1.2	50.6 ± 1.9	29.8 ± 1.1	55.6 ± 1.9
**A8**	31.8 ± 2.6	51.6 ± 1.8	35.6 ± 1.2	48.9 ± 1.3	30.3 ± 2.9	45.9 ± 1.7
**A9**	40.8 ± 2.3	59.2 ± 1.9	45.2 ± 1.8	61.4 ± 2.5	37.9 ± 1.1	54.8 ± 1.9
**A10**	38.9 ± 1.2	54.9 ± 3.1	43.3 ± 2.4	57.2 ± 1.9	35.6 ± 2.0	51.7 ± 2.2
**A11**	53.6 ± 2.6	67.9 ± 1.8	53.6 ± 3.1	65.6 ± 2.5	47.2 ± 2.7	63.8 ± 1.9
**A12**	34.8 ± 2.8	49.7 ± 1.1	30.9 ± 2.1	56.5 ± 1.8	33.1 ± 1.4	43.9 ± 1.3
**A13**	38.9 ± 1.5	62.1 ± 2.5	40.8 ± 1.6	57.6 ± 2.3	36.5 ± 2.4	56.5 ± 2.1
**A14**	33.8 ± 1.8	43.7 ± 1.7	31.3 ± 2.8	46.5 ± 0.9	33.7 ± 2.0	40.0 ± 0.8
**A15**	43.3 ± 2.1	51.9 ± 2.8	40.1 ± 2.2	63.1 ± 3.3	33.0 ± 1.1	43.6 ± 1.9
**A16**	57.2 ± 2.4	68.2 ± 1.6	56.5 ± 1.9	71.8 ± 2.9	48.3 ± 1.6	69.5 ± 2.8
**A17**	39.3 ± 1.9	61.2 ± 2.2	41.2 ± 2.1	60.5 ± 3.1	33.9 ± 2.7	54.2 ± 1.9
**A18**	52.3 ± 2.6	65.2 ± 1.9	55.6 ± 1.2	70.2 ± 2.9	47.9 ± 1.1	65.6 ± 2.5
**A19**	36.8 ± 1.7	53.1 ± 2.4	31.9 ± 1.0	51.8 ± 1.7	29.0 ± 1.5	43.7 ± 1.9
**A20**	51.3 ± 2.7	67.1 ± 2.3	51.1 ± 2.4	68.1 ± 2.6	48.7 ± 1.9	62.8 ± 1.3
**A21**	47.3 ± 2.2	60.0 ± 1.9	50.3 ± 3.0	61.7 ± 1.3	45.3 ± 2.8	55.2 ± 2.6
**B1**	31.5 ± 1.8	45.3 ± 2.1	28.6 ± 1.3	46.2 ± 2.5	27.3 ± 1.9	37.5 ± 2.1
**B2**	30.4 ± 2.5	48.9 ± 2.3	29.3 ± 1.8	43.5 ± 0.9	31.1 ± 1.5	41.8 ± 1.2
**B3**	32.8 ± 1.9	46.7 ± 1.3	35.6 ± 3.2	45.1 ± 1.7	33.9 ± 2.4	44.6 ± 1.8
**B4**	36.7 ± 2.3	52.1 ± 2.6	38.5 ± 1.9	58.4 ± 2.2	32.8 ± 1.4	46.9 ± 3.1
**B5**	40.8 ± 1.7	43.4 ± 3.9	36.3 ± 2.1	50.6 ± 3.3	33.0 ± 1.6	42.6 ± 1.8
**B6**	26.4 ± 1.9	41.9 ± 2.3	28.1 ± 1.7	43.5 ± 2.2	23.9 ± 2.8	43.0 ± 2.1
**B7**	42.9 ± 1.2	43.1 ± 1.2	41.2 ± 0.9	50.1 ± 1.8	36.6 ± 1.2	52.9 ± 2.4
**B8**	29.5 ± 2.6	46.7 ± 2.7	38.1 ± 1.4	43.6 ± 3.1	28.9 ± 2.1	39.6 ± 1.1
**B9**	42.4 ± 1.9	54.1 ± 3.1	45.1 ± 1.5	58.8 ± 2.8	38.0 ± 1.8	52.1 ± 3.4
**B10**	40.6 ± 2.5	51.4 ± 3.2	38.5 ± 2.2	41.8 ± 1.1	30.3 ± 1.7	43.9 ± 1.6
**B11**	43.6 ± 1.0	58.9 ± 1.9	52.9 ± 3.7	63.7 ± 1.9	42.8 ± 2.0	60.5 ± 1.3
**B12**	29.8 ± 1.4	46.8 ± 2.5	35.2 ± 1.2	49.1 ± 2.0	32.8 ± 1.7	41.9 ± 2.2
**B13**	40.1 ± 2.6	51.9 ± 1.1	30.5 ± 1.6	55.4 ± 2.1	36.1 ± 2.8	48.1 ± 2.9
Ribavirin^b^	39.9 ± 2.3	51.2 ± 1.2	35.6 ± 1.6	48.8 ± 1.9	37.8 ± 1.0	46.2 ± 2.1
Ningnanmycin^b^	49.8 ± 1.8	62.3 ± 2.5	51.8 ± 3.1	63.3 ± 1.7	49.1 ± 2.8	65.2 ± 1.7

a Average of three replicates.

b The commercial antiviral agents ribavirin and ningnanmycin were used for comparison of activity.

**TABLE 5:** The EC_50_ values of the compounds against TMV, PMMoV, and TSWV^a^.

Compd.	TMV		PMMoV		TSWV	
	Curative activity	Protective activity	Curative activity	Protective activity	Curative activity	Protective activity
**A4**	501.4 ± 6.3	289.5 ± 4.8	482.7 ± 7.9	196.5 ± 5.8	601.4 ± 9.5	312.1 ± 8.4
**A11**	489.5 ± 9.0	225.8 ± 9.1	491.3 ± 5.8	219.6 ± 4.9	585.3 ± 7.4	354.2 ± 9.0
**A16**	368.7 ± 3.3	210.4 ± 8.8	310.8 ± 9.1	156.2 ± 8.1	576.9 ± 3.7	178.9 ± 3.1
**A18**	410.5 ± 5.9	251.2 ± 7.1	345.6 ± 3.4	178.2 ± 3.6	610.4 ± 3.8	215.2 ± 6.2
**A20**	490.2 ± 8.5	301.5 ± 6.2	411.9 ± 5.7	270.3 ± 4.7	595.2 ± 5.2	380.5 ± 9.1
**B11**	560.2 ± 4.9	318.9 ± 6.6	426.3 ± 9.1	280.5 ± 3.6	610.4 ± 5.8	368.1 ± 4.6
Ribavirin^b^	690.5 ± 7.5	505.1 ± 4.6	780.5 ± 8.6	568.6 ± 5.6	810.7 ± 9.2	650.2 ± 4.5
Ningnanmycin^b^	420.5 ± 6.5	242.6 ± 7.7	415.8 ± 4.9	218.4 ± 6.3	408.8 ± 8.1	180.5 ± 3.9

a Average of three replicates.

b The commercial antiviral agents ribavirin and ningnanmycin were used for comparison of activity.

Structure-activity relationship analysis based on protective activity against three viruses indicates that **A** series compounds have better antiviral activity than **B** series compounds, which is consistent with the trend of nematicidal activity. Further structure-activity relationship analysis demonstrated that the compound with R_1_ as OCH_3_ showed better antiviral activity than that of the compounds with other groups, such as **A13** (R^1^ = 4-OCH_3_, R^2^ = H) > **A21** (R^1^ = 4-Br, R^2^ = H), **A10** (R^1^ = 4-CH_3_, R^2^ = H), **A5** (R^1^ = H, R^2^ = H), and **A1** (R^1^ = 2,4-diCl, R^2^ = H). In particular, the compound with 2-OCH_3_ of R^1^ had the best antiviral activity; for example, **A16** (R^1^ = 2-OCH_3_, R^2^ = H) > **A13** (R^1^ = 4-OCH_3_, R^2^ = H) > **A14** (R^1^ = 3-OCH_3_, R^2^ = H). Compared with the electron-donating group (CH_3_), the introduction of strong electron withdraw group (F) into R_2_ can favor antiviral activity, such as **A18** (R^1^ = 2-OCH_3_, R^2^ = 4-F) > **A17** (R^1^ = 2-OCH_3_, R^2^ = 4-CH_3_) and **A11** (R^1^ = 4-OCH_3_, R^2^ = 4-F) > **A12** (R^1^ = 4-OCH_3_, R^2^ = 4-CH_3_), **A4** (R^1^ = 2,4-diCl, R^2^ = 4-F) > **A2** (R^1^ = 2,4-diCl, R^2^ = 4-CH_3_).

## Conclusion

In the present work, 34 novel chalcone derivatives containing an 1,2,4-oxadiazole moiety were synthesized and assessed for the nematocidal and antiviral activities of all of the compounds. The results show that compounds **A13** and **A14** have excellent nematocidal activities against *B. xylophilus*, *A. besseyi*, and *D. dipsaci* and are superior to tioxazafen, fosthiazate, and abamectin. Furthermore, compound **A16** has better protective activity against TMV, PMMoV, and TSWV than that of ribavirin and ningnanmycin. Therefore, chalcone derivatives containing an 1,2,4-oxadiazole moiety can be considered as candidate leading structures for the development of new pesticides.

## Data Availability

The datasets presented in this study can be found in online repositories. The names of the repository/repositories and accession number(s) can be found in the article/Supplementary Material.
