# Supplementary material for: Design, Synthesis, and Biological Activity of Novel Chalcone Derivatives Containing an 1,2,4-Oxadiazole Moiety
Source: Front Chem. 2022 Jul 22;10:943062. doi: 10.3389/fchem.2022.943062 (PMC9354253; doi:10.3389/fchem.2022.943062)
Supplement: Supplementary file 1 [file DataSheet1.doc]

**TABLE 1.** The reaction conditions for the compound **A1** were optimized

| Entry | Catalyst | Solvent | Temperature/◦C | Yield^a^/% |
| --- | --- | --- | --- | --- |
| 1 | K_2_CO_3_ | CH_3_CN | r.t. | 32% |
| 2 | Na_2_CO_3_ | CH_3_CN | r.t | 15% |
| 3 | NaOH | CH_3_CN | r.t | 21% |
| 3 | K_2_CO_3_/KI | CH_3_CN | r.t | 38% |
| 4 | K_2_CO_3_/KI | CH_3_CN | 80 | 71% |
| 5 | K_2_CO_3_ | DMF | r.t | 39% |
| 6 | K_2_CO_3_/KI | DMF | r.t | 56% |
| 7 | K_2_CO_3_ | DMF | 60 | 85% |
| 8 | K_2_CO_3_ | (CH_3_)_2_CO | 56 | 48% |
| 9 | K_2_CO_3_ | DMF | 80 | 83% |
| 10 | K_2_CO_3_/KI | DMF | 60 | 89% |

^a^Isolated yield.

**TABLE 2.** Nematicidal activity of compounds **A1**−**A21** and **B1**−**B13***^a.^*

| Compd. | Corrected Mortality ± SD (%) ***^b^*** | | | | | |
| --- | --- | --- | --- | --- | --- | --- |
|  | *B. xylophilus* | | *A. besseyi* | | *D. dipsaci* | |
|  | 50 *µ*g/mL | 10 *µ*g/mL | 50 *µ*g/mL | 10 *µ*g/mL | 50 *µ*g/mL | 10 *µ*g/mL |
| **A1** | 26.6 ± 3.8 | - | 32.0 ± 6.5 | - | - | - |
| **A2** | - | - | - | - | 22.4 ± 2.9 | - |
| **A3** | - | - | - | - | - | - |
| **A4** | 37.7 ± 3.9 | - | - | - | - | - |
| **A5** | 38.8 ± 4.9 | - | 32.0 ± 6.5 | - | 23.1 ± 1.3 | - |
| **A6** | 30.6 ± 5.8 | - | 21.2 ± 2.4 | - | 20.6 ± 5.2 | - |
| **A7** | - | - | 24.6 ± 5.3 | 20.5 ± 2.2 | 24.7 ± 0.4 | - |
| **A8** | - | - | 21.5 ± 2.3 | - | 22.6 ± 8.0 | - |
| **A9** | 48.9 ± 7.4 | 26.9 ± 5.7 | - | - | 30.7 ± 3.5 | 24.2 ± 5.9 |
| **A10** | 26.9 ± 5.2 | - | - | - | 30.7 ± 5.3 | - |
| **A11** | 20.7 ± 4.7 | - | 24.1 ± 8.6 | - | 22.0 ± 2.8 | - |
| **A12** | 41.0 ± 6.9 | 23.5 ± 4.1 | - | - | 27.7 ± 8.2 | - |
| **A13** | 100 | 25.8 ± 4.9 | 100 | 25.8 ± 5.9 | 100 | 25.8 ± 1.9 |
| **A14** | 100 | 25.1 ± 5.6 | 100 |  | 100 | 24.1 ± 1.6 |
| **A15** | 47.9 ± 5.7 | 32.1 ± 7.7 | - | - | - | - |
| **A16** | 21.5 ± 1.5 | - | - | - | - | - |
| **A17** | - | - | - | - | - | - |
| **A18** | 36.5 ± 9.5 | 25.4 ± 1.0 | - | - | 25.4 ± 1.0 | - |
| **A19** | 44.9 ± 6.1 | 23.3 ± 6.7 | - | - | - | - |
| **A20** | 25.5 ± 5.5 | - | - | - | - | - |
| **A21** | 23.0 ± 4.5 | - | - | - | 23.4 ± 6.2 | - |
| **B1** | 29.9 ± 6.5 | - | 25.2 ± 7.1 | - | 22.4 ± 6.0 | - |
| **B2** | 37.7 ± 6.7 | - | 21.8 ± 3.3 | - | 28.8 ± 1.2 | - |
| **B3** | 51.8 ± 5.8 | - | 25.6 ± 4.0 | - | 25.5 ± 3.8 | - |
| **B4** | 28.1 ± 7.8 | - | 25.0 ± 3.3 | - | 24.9 ± 9.0 | - |
| **B5** | 31.1 ± 5.8 | - | 29.5 ± 2.8 | 20.8 ± 7.3 | 33.2 ± 1.5 | 23.3 ± 2.7 |
| **B6** | 37.0 ± 1.1 | - | 70.8 ± 1.8 | - | 29.9 ± 6.2 | 20.7 ± 6.6 |
| **B7** | 27.8 ± 6.3 | - | - | - | 21.6 ± 4.4 | - |
| **B8** | - | - | - | - | 25.3 ± 5.9 | - |
| **B9** | - | - | - | - | 29.3 ± 3.4 | - |
| **B10** | - | - | 22.9 ± 2.8 | - | 22.4 ± 4.7 | - |
| **B11** | - | - | 31.5 ± 4.3 | - | 41.0 ± 7.4 | 22.2 ± 4.1 |
| **B12** | - | - | 59.6 ± 9.2 | - | - | - |
| **B13** | 25.1 ± 1.1 | - | 35.8 ± 1.7 | - | 33.0 ± 6.5 | - |
| **Tioxazafen***^b^* | 34.3 ± 7.7 | - | 40.0 ± 6.1 | 20.1 ± 2.5 | 29.0 ± 3.7 | - |
| **Fosthiazate***^b^* | 43.9 ± 5.2 | 23.2 ± 9.8 | - | - | 33.3 ± 1.6 | - |
| **Abamectin***^b^* | 49.4 ± 6.3 | 31.9 ± 4.2 | 42.3 ± 2.0 | 22.2 ± 3.2 | 33.6 ± 1.3 | 20.2 ± 3.3 |

*^a^*Average of three replicates. *^b^*The commercial antiviral agent tioxazafen, fosthiazate, and abamectin were used for comparison of activity. “-” No activity or corrected mortality < 20%.

**TABLE 3.** The LC_50_ values of nematicidal activity of compounds.

| Compd. | LC_50_ (*μ*g/mL) *^a^* | | |
| --- | --- | --- | --- |
|  | *B. xylophilus* | *A. besseyi* | *B. cinerea* |
| **A13** | 35.5 ± 3.5 | 44.7 ± 5.4 | 30.2 ± 2.0 |
| **A14** | 31.8 ± 0.9 | 47.4 ± 2.5 | 36.5 ± 0.7 |
| **Tioxazafen***^b^* | > 200 | > 200 | > 200 |
| **Fosthiazate***^b^* | > 200 | > 200 | > 200 |
| **Abamectin***^b^* | 103.8 ± 1.5 | > 200 | 106.2 ± 2.1 |

*^a^*Average of three replicates. *^b^*The commercial antiviral agent tioxazafen, fosthiazate, and abamectin were used for comparison of activity.

**TABLE 4**. Antiviral activities of compounds **A1**−**A21**and **B1**−**B13** at 500 *µ*g/mL*a*

| Compd. | TMV | | | | PMMoV | | | | TSWV | | | |  |
| --- | --- | --- | --- | --- | --- | --- | --- | --- | --- | --- | --- | --- | --- |
|  | Curative  activity (%) | | Protective activity (%) | | Curative  activity (%) | | | Protective activity (%) | Curative  activity (%) | | Protective activity (%) | |  |
| **A1** | | 45.6 ± 1.9 | | 60.3 ± 2.5 | | 39.5 ± 1.1 | 56.1 ± 1.8 | | | 27.8 ± 3.0 | | 46.5 ± 2.2 | |
| **A2** | | 38.9 ± 2.9 | | 49.8 ± 1.1 | | 45.3 ± 2.5 | 57.2 ± 1.4 | | | 35.7 ± 1.0 | | 45.6 ± 2.3 | |
| **A3** | | 36.1 ± 2.3 | | 47.2 ± 2.6 | | 40.6 ± 1.7 | 49.3 ± 1.8 | | | 32.9 ± 2.7 | | 48.0 ± 1.9 | |
| **A4** | | 49.8 ± 1.1 | | 64.5 ± 3.4 | | 52.3 ± 2.5 | 67.1 ± 2.3 | | | 46.7 ± 1.9 | | 63.1 ± 2.8 | |
| **A5** | | 23.6 ± 2.6 | | 54.2 ± 1.9 | | 39.8 ± 1.9 | 60.2 ± 2.2 | | | 31.2 ± 1.3 | | 54.8 ± 2.9 | |
| **A6** | | 37.8 ± 2.1 | | 54.1 ± 2.9 | | 43.8 ± 3.1 | 59.2 ± 3.1 | | | 33.3 ± 1.7 | | 51.2 ± 2.5 | |
| **A7** | | 30.6 ± 1.8 | | 49.5 ± 2.5 | | 36.3 ± 1.2 | 50.6 ± 1.9 | | | 29.8 ± 1.1 | | 55.6 ± 1.9 | |
| **A8** | | 31.8 ± 2.6 | | 51.6± 1.8 | | 35.6 ± 1.2 | 48.9 ± 1.3 | | | 30.3 ± 2.9 | | 45.9 ± 1.7 | |
| **A9** | | 40.8 ± 2.3 | | 59.2± 1.9 | | 45.2 ± 1.8 | 61.4 ± 2.5 | | | 37.9 ± 1.1 | | 54.8 ± 1.9 | |
| **A10** | | 38.9 ± 1.2 | | 54.9 ± 3.1 | | 43.3 ± 2.4 | 57.2 ± 1.9 | | | 35.6 ± 2.0 | | 51.7 ± 2.2 | |
| **A11** | | 53.6 ± 2.6 | | 67.9 ±1.8 | | 53.6 ± 3.1 | 65.6 ± 2.5 | | | 47.2 ± 2.7 | | 63.8 ± 1.9 | |
| **A12** | | 34.8 ± 2.8 | | 49.7 ± 1.1 | | 30.9 ± 2.1 | 56.5 ± 1.8 | | | 33.1 ± 1.4 | | 43.9 ± 1.3 | |
| **A13** | | 38.9 ± 1.5 | | 62.1 ± 2.5 | | 40.8 ± 1.6 | 57.6 ± 2.3 | | | 36.5 ± 2.4 | | 56.5 ± 2.1 | |
| **A14** | | 33.8 ± 1.8 | | 43.7 ± 1.7 | | 31.3 ± 2.8 | 46.5 ± 0.9 | | | 33.7 ± 2.0 | | 40.0 ± 0.8 | |
| **A15** | | 43.3 ± 2.1 | | 51.9 ± 2.8 | | 40.1 ± 2.2 | 63.1 ± 3.3 | | | 33.0 ± 1.1 | | 43.6 ± 1.9 | |
| **A16** | | 57.2 ± 2.4 | | 68.2 ± 1.6 | | 56.5 ± 1.9 | 71.8 ± 2.9 | | | 48.3 ± 1.6 | | 69.5 ± 2.8 | |
| **A17** | | 39.3 ± 1.9 | | 61.2 ± 2.2 | | 41.2 ± 2.1 | 60.5 ± 3.1 | | | 33.9 ± 2.7 | | 54.2 ± 1.9 | |
| **A18** | | 52.3 ± 2.6 | | 65.2 ± 1.9 | | 55.6 ± 1.2 | 70.2 ± 2.9 | | | 47.9 ± 1.1 | | 65.6 ± 2.5 | |
| **A19** | | 36.8 ± 1.7 | | 53.1 ± 2.4 | | 31.9 ± 1.0 | 51.8 ± 1.7 | | | 29.0 ± 1.5 | | 43.7 ± 1.9 | |
| **A20** | | 51.3 ± 2.7 | | 67.1 ± 2.3 | | 51.1 ± 2.4 | 68.1 ± 2.6 | | | 48.7 ± 1.9 | | 62.8 ± 1.3 | |
| **A21** | | 47.3 ± 2.2 | | 60.0 ± 1.9 | | 50.3 ± 3.0 | 61.7 ± 1.3 | | | 45.3 ± 2.8 | | 55.2 ± 2.6 | |
| **B1** | | 31.5 ± 1.8 | | 45.3 ± 2.1 | | 28.6 ± 1.3 | 46.2 ± 2.5 | | | 27.3 ± 1.9 | | 37.5 ± 2.1 | |
| **B2** | | 30.4 ± 2.5 | | 48.9 ± 2.3 | | 29.3 ± 1.8 | 43.5 ± 0.9 | | | 31.1 ± 1.5 | | 41.8 ± 1.2 | |
| **B3** | | 32.8 ± 1.9 | | 46.7 ± 1.3 | | 35.6 ± 3.2 | 45.1 ± 1.7 | | | 33.9 ± 2.4 | | 44.6 ± 1.8 | |
| **B4** | | 36.7 ± 2.3 | | 52.1 ± 2.6 | | 38.5 ± 1.9 | 58.4 ± 2.2 | | | 32.8 ± 1.4  33.0 ± 1.6 | | 46.9 ± 3.1 | |
| **B5** | | 40.8 ± 1.7 | | 43.4 ± 3.9 | | 36.3 ± 2.1 | 50.6 ± 3.3 | | |  |  | 42.6 ± 1.8 | |
| **B6** | | 26.4 ± 1.9 | | 41.9 ± 2.3 | | 28.1 ± 1.7 | 43.5 ± 2.2 | | | 23.9 ± 2.8 | | 43.0 ± 2.1 | |
| **B7** | | 42.9 ± 1.2 | | 43.1 ± 1.2 | | 41.2 ± 0.9 | 50.1 ± 1.8 | | | 36.6 ± 1.2 | | 52.9 ± 2.4 | |
| **B8** | | 29.5 ± 2.6 | | 46.7 ± 2.7 | | 38.1 ± 1.4 | 43.6 ± 3.1 | | | 28.9 ± 2.1 | | 39.6 ± 1.1 | |
| **B9** | | 42.4 ± 1.9 | | 54.1 ± 3.1 | | 45.1 ± 1.5 | 58.8 ± 2.8 | | | 38.0 ± 1.8 | | 52.1 ± 3.4 | |
| **B10** | | 40.6 ± 2.5 | | 51.4 ± 3.2 | | 38.5 ± 2.2 | 41.8 ± 1.1 | | | 30.3 ± 1.7 | | 43.9 ± 1.6 | |
| **B11** | | 43.6 ± 1.0 | | 58.9 ± 1.9 | | 52.9 ± 3.7 | 63.7 ± 1.9 | | | 42.8 ± 2.0 | | 60.5 ± 1.3 | |
| **B12** | | 29.8 ± 1.4 | | 46.8 ± 2.5 | | 35.2 ± 1.2 | 49.1 ± 2.0 | | | 32.8 ± 1.7 | | 41.9 ± 2.2 | |
| **B13** | | 40.1 ± 2.6 | | 51.9 ± 1.1 | | 30.5 ± 1.6 | 55.4 ± 2.1 | | | 36.1 ± 2.8 | | 48.1 ± 2.9 | |
| Ribavirin*^b^* | | 39.9 ± 2.3 | | 51.2 ± 1.2 | | 35.6 ± 1.6 | 48.8 ± 1.9 | | | 37.8 ± 1.0 | | 46.2 ± 2.1 | |
| Ningnanmycin*^b^* | | 49.8 ± 1.8 | | 62.3 ± 2.5 | | 51.8 ± 3.1 | 63.3± 1.7 | | | 49.1 ± 2.8 | | 65.2 ± 1.7 | |

*^a^*Average of three replicates. *^b^*The commercial antiviral agent ribavirin and ningnanmycin were used for comparison of activity.

**TABLE 5**. The EC50 values of the compounds against TMV, PMMoV and TSWV *a*

| Compd. | TMV | | PMMoV | | | TSWV | |
| --- | --- | --- | --- | --- | --- | --- | --- |
|  | Curative  activity | Protective activity | | Curative  activity | Protective activity | Curative  activity | Protective activity |
| **A4** | 501.4 ±6.3 | 289.5 ± 4.8 | | 482.7 ± 7.9 | 196.5 ± 5.8 | 601.4 ± 9.5 | 312.1 ± 8.4 |
| **A11** | 489.5 ± 9.0 | 225.8 ±9.1 | | 491.3 ± 5.8 | 219.6 ± 4.9 | 585.3 ± 7.4 | 354.2 ± 9.0 |
| **A16** | 368.7 ± 3.3 | 210.4 ± 8.8 | | 310.8 ± 9.1 | 156.2 ± 8.1 | 576.9 ± 3.7 | 178.9 ± 3.1 |
| **A18** | 410.5 ± 5.9 | 251.2 ± 7.1 | | 345.6 ± 3.4 | 178.2 ± 3.6 | 610.4 ± 3.8 | 215.2 ± 6.2 |
| **A20** | 490.2 ± 8.5 | 301.5 ± 6.2 | | 411.9 ± 5.7 | 270.3 ± 4.7 | 595.2 ± 5.2 | 380.5 ± 9.1 |
| **B11** | 560.2 ± 4.9 | 318.9 ± 6.6 | | 426.3 ± 9.1 | 280.5 ± 3.6 | 610.4 ± 5.8 | 368.1 ± 4.6 |
| Ribavirin*^b^* | 690.5 ± 7.5 | 505.1 ± 4.6 | | 780.5 ± 8.6 | 568.6 ± 5.6 | 810.7 ± 9.2 | 650.2± 4.5 |
| Ningnanmycin*^b^* | 420.5 ± 6.5 | 242.6 ± 7.7 | | 415.8 ± 4.9 | 218.4± 6.3 | 408.8 ± 8.1 | 180.5 ± 3.9 |

*^a^*Average of three replicates. *^b^* The commercial antiviral agent ribavirin and ningnanmycin were used for comparison of activity.
