# Supplementary material for: Design, Synthesis, and Biological Activity of Novel Chalcone Derivatives Containing an 1,2,4-Oxadiazole Moiety
Source: Front Chem. 2022 Jul 22;10:943062. doi: 10.3389/fchem.2022.943062 (PMC9354253; doi:10.3389/fchem.2022.943062)
Supplement: Supplementary file 2 [file DataSheet2.doc]

**Supporting Information**

Design, synthesis, biological activity of novel 1,2,4-oxadiazole derivatives containing chalcone moiety

Ling Luo^a^, Dan Liu^a^, Shichao Lan^a, b^, Xiuhai Gan^a^*

*^a^State Key Laboratory Breeding Base of Green Pesticide and Agricultural Bioengineering, Key Laboratory of Green Pesticide and Agricultural Bioengineering, Ministry of Education, Guizhou University, Huaxi District, Guiyang 550025, China*

*^b^School of Biological Sciences, Guizhou Education University, Guiyang 550018, PR China*

*The address of the correspondence author: li

Fax: 0086-851-83622211, E-mail: gxh200719@163.com

**Contents**

1.Structures and characterization data of target compounds **A1**‒**A21** and **B1**‒**B13**

2. ^1^H NMR, ^13^C NMR, and HRMS spectra of target compounds **A1‒A21** and **B1‒B13**

**1. Structures and characterization data of target compounds A1‒A21 and B1‒B13**

(*E*)-3-(2,4-dichlorophenyl)-1-(4-((3-phenyl-1,2,4-oxadiazol-5-yl)methoxy)phenyl)prop-2-en-1-one (**A1**). White powder; m.p. 154-155˚C; yield 89%; ^1^H NMR (400 MHz, DMSO-*d_6_*): *δ* 8.02 (d, *J* = 6.4 Hz, 2H), 7.79 (d, *J* = 8.8 Hz, 2H), 7.77 (d, *J* = 1.6 Hz, 1H), 7.59-7.58 (m, 5H), 7.40 (d, *J* = 16.0 Hz, 1H), 7.17 (d, *J* = 9.2 Hz, 2H), 7.16 (d, *J* = 15.6 Hz, 1H), 5.70 (s, 2H); ^13^C NMR (101 MHz, DMSO-*d_6_*): *δ* 192.83, 175.99, 168.85, 160.07, 147.02, 138.07, 135.90, 132.31, 131.63, 131.50, 131.50, 131.10, 130.12, 129.84, 129.84, 128.38, 128.10, 127.56, 127.56, 126.20, 124.95, 115.80, 115.80, 61.39. HRMS (ESI) m/z for C_24_H_17_O_3_N_2_Cl_2_ [M+H]^+^ calcd: 451.06107, found: 451.05978.

(*E*)-3-(2,4-dichlorophenyl)-1-(4-((3-(p-tolyl)-1,2,4-oxadiazol-5-yl)methoxy)phenyl)prop-2-en-1-one (**A2**). Faint yellow powder; m.p. 151-152˚C; yield 94%; ^1^H NMR (400 MHz, DMSO-*d_6_*): *δ* 7.90 (d, *J* = 8.4 Hz, 2H), 7.79 (d, *J* = 8.8 Hz, 2H), 7.76 (d, *J* = 1.6 Hz, 1H), 7.59 (d, *J* = 16.0 Hz, 1H), 7.56 (d, *J* = 8.0, 1.6 Hz, 1H), 7.39 (d, *J* = 15.6 Hz, 1H), 7.37 (d, *J* = 6.4 Hz, 2H), 7.17 (d, *J* = 2.0 Hz, 2H), 7.14 (*J* = 5.2 Hz, 1H), 5.68 (s, 2H), 2.38 (s, 3H); ^13^C NMR (101 MHz, DMSO-*d_6_*): *δ* 193.19, 176.28, 167.52, 160.43, 147.93, 142.47, 137.73, 135.90, 131.63, 131.50, 131.10, 130.38, 130.38, 130.11, 128.38, 128.11, 127.50, 127.50, 124.94, 123.43, 115.79, 115.79, 61.07, 21.57. HRMS (ESI) m/z for C_25_H_19_O_3_N_2_Cl_2_ [M+H]^+^ calcd: 465.07672, found: 465.07529.

(*E*)-1-(4-((3-(4-chlorophenyl)-1,2,4-oxadiazol-5-yl)methoxy)phenyl)-3-(2,4-dichlorophenyl)prop-2-en-1-one (**A3**). White powder; m.p. 152-153˚C; yield 90%; ^1^H NMR (400 MHz, DMSO-*d_6_*): *δ* 8.02 (d, *J* = 8.4 Hz, 2H), 7.81-7.78 (m, 3H), 7.65 (d, *J* = 8.4 Hz, 2H), 7.59-7.58 (m, 2H), 7.40 (d, *J* = 16.0 Hz, 1H), 7.17 (d, *J* = 8.8 Hz, 2H), 7.16 (d, *J* = 15.6 Hz, 1H), 5.70 (s, 2H); ^13^C NMR (101 MHz, DMSO-*d_6_*): *δ* 192.83, 176.24, 167.47, 160.04, 147.00, 138.06, 137.03, 135.90, 131.63, 131.49, 131.49, 131.10, 130.11, 130.02, 130.02, 129.37, 129.37, 128.39, 128.10, 125.06, 124.96, 115.79, 115.79, 61.38. HRMS (ESI) m/z for C_24_H_16_O_3_N_2_Cl_3_ [M+H]^+^ calcd: 485.02210, found: 485.02072.

(*E*)-1-(4-((3-(4-fluorophenyl)-1,2,4-oxadiazol-5-yl)methoxy)phenyl)-3-(2,4-dichlorophenyl)prop-2-en-1-one (**A4**). White powder; m.p. 148-149˚C; yield 93%; ^1^H NMR (400 MHz, DMSO-*d*_6_): *δ* 8.09-8.05 (m, 2H), 7.79 (d, *J* = 7.2 Hz, 2H), 7.77 (d, *J* = 2.0 Hz, 1H), 7.61-7.56 (m, 2H), 7.43 (d, *J* = 8.8 Hz, 2H), 7.40 (d, *J* = 15.2 Hz, 1H), 7.18 (d, *J* = 2.0 Hz, 2H), 7.17 (d, *J* = 16.0 Hz, 1H), 7.14 (*J* = 5.2 Hz, 1H), 5.69 (s, 2H); ^13^C NMR (101 MHz, DMSO-*d*_6_): *δ* 192.82, 176.10, 167.45, 165.50, 160.05, 147.00, 138.06, 135.90, 131.63, 131.49, 131.49, 131.10, 130.20, 130.11, 130.11, 128.39, 128.10, 124.95, 122.79, 122.77, 117.14, 116.92, 115.79,115.79, 61.37. HRMS (ESI) m/z for C_24_H_16_O_3_N_2_Cl_2_F [M+H]^+^ calcd: 469.05165, found: 469.05026.

(*E*)-3-phenyl-1-(4-((3-phenyl-1,2,4-oxadiazol-5-yl)methoxy)phenyl)prop-2-en-1-one (**A5**). White powder; m.p. 155-156˚C; yield 53%; ^1^H NMR (400 MHz, DMSO-*d*_6_): *δ* 8.21 (d, *J* = 9.2 Hz, 2H), 7.96 (d, *J* = 15.6 Hz, 1H), 7.92 (d, *J* = 8.4 Hz, 2H), 7.90-7.88 (m, 2H), 7.73 (d, *J* = 15.6 Hz, 1H), 7.47-7.46 (m, 3H), 7.39 (d, *J* = 8.0 Hz, 2H), 7.27 (d, *J* = 8.8 Hz, 2H), 5.75 (s, 2H), 2.39 (s, 3H); ^13^C NMR (101 MHz, DMSO-*d*_6_): *δ* 187.92, 175.91, 168.27, 161.54, 143.93, 135.23, 132.31, 132.03, 131.47, 131.47, 130.98, 129.84, 129.84, 129.37, 129.37, 129.32, 129.32, 127.57, 127.57, 126.20, 122.43, 115.32, 115.32, 61.49. HRMS (ESI) m/z for C_24_H_19_O_3_N_2_ [M+H]^+^ calcd: 383.13902, found: 383.13797.

(*E*)-3-phenyl-1-(4-((3-(p-tolyl)-1,2,4-oxadiazol-5-yl)methoxy)phenyl)prop-2-en-1-one (**A6**). White powder; m.p. 175-176˚C; yield 39%; ^1^H NMR (400 MHz, DMSO-*d*_6_): *δ* 8.26 (d, *J* = 8.8 Hz, 2H), 8.07 (dd, *J* = 8.0, 1.6 Hz, 2H), 8.00 (d, *J* = 15.6 Hz, 1H), 7.94-7.92 (m, 2H), 7.77 (d, *J* = 15.6 Hz, 1H), 7.65-7.63 (m, 3H), 7.51-7.49 (m, 3H), 7.31 (d, *J* = 9.2 Hz, 2H), 5.81 (s, 2H); ^13^C NMR (101 MHz, DMSO-*d*_6_): *δ* 187.92, 175.73, 168.23, 161.55, 143.93, 142.31, 135.23, 132.02, 131.47, 131.47, 130.99, 130.39, 130.39, 129.37, 129.37, 129.32, 129.32, 127.51, 127.51, 123.42, 122.43, 115.31, 115.31, 61.48, 21.57. HRMS (ESI) m/z for C_25_H_21_O_3_N_2_ [M+H]^+^ calcd: 397.15467, found: 397.15359.

(*E*)-1-(4-((3-(4-chlorophenyl)-1,2,4-oxadiazol-5-yl)methoxy)phenyl)-3-phenylprop-2-en-1-one (**A7**). White powder; m.p. 180-181˚C; yield 60%; ^1^H NMR (400 MHz, DMSO-*d*_6_): *δ* 8.22 (d, *J* = 8.8 Hz, 2H), 8.04 (d, *J* = 8.4 Hz, 2H), 7.97 (d, *J* = 15.6 Hz, 1H), 7.90-7.88 (m, 2H), 7.73 (d, *J* = 15.6 Hz, 1H), 7.66 (d, *J* = 8.8 Hz, 2H), 7.47-7.46 (m, 3H), 7.27 (d, *J* = 8.8 Hz, 2H), 5.77 (s, 2H); ^13^C NMR (101 MHz, DMSO-*d*_6_): *δ* 187.78, 176.30, 166.82, 161.39, 143.86, 137.02, 135.16, 131.91, 131.41, 131.41, 130.90, 130.04, 130.04, 129.38, 129.38, 129.33, 129.33, 129.19, 129.19, 125.00, 122.36, 115.28, 115.28, 61.42. HRMS (ESI) m/z for C_24_H_18_O_3_N_2_Cl [M+H]^+^ calcd: 417.10005, found: 417.09875.

(*E*)-1-(4-((3-(4-fluorophenyl)-1,2,4-oxadiazol-5-yl)methoxy)phenyl)-3-phenylprop-2-en-1-one (**A8**). White powder; m.p. 168-169˚C; yield 66%; ^1^H NMR (400 MHz, DMSO-*d*_6_): *δ* 8.22 (d, *J* = 8.8 Hz, 2H), 8.10-8.07 (m, 2H), 7.96 (d, *J* = 15.6 Hz, 1H), 7.90-7.88 (m, 2H), 7.73 (d, *J* = 15.6 Hz, 1H), 7.47-7.41 (m, 5H), 7.27 (d, *J* = 8.8 Hz, 2H), 5.77 (s, 2H); ^13^C NMR (101 MHz, DMSO-*d*_6_): *δ* 187.92, 176.03, 167.49, 165.67 (C-F), 163.32 (C-F), 161.52, 143.93, 135.23, 132.04, 131.47, 131.47, 130.98, 130.22, 130.13, 129.37, 129.37, 129.32, 129.32, 122.77, 122.42, 117.15, 116.93, 115.31, 115.31 61.47. HRMS (ESI) m/z for C_24_H_18_O_3_N_2_F [M+H]^+^ calcd: 401.12960, found: 401.12845.

(*E*)-3-(2-chlorophenyl)-1-(4-((3-(4-chlorophenyl)-1,2,4-oxadiazol-5-yl)methoxy)phenyl)prop-2-en-1-one (**A9**). White powder; m.p. 190-191˚C; yield 71%; ^1^H NMR (600 MHz, DMSO-*d*_6_): *δ* 8.23-8.21 (m, 3H), 8.04-8.01 (m, 4H), 7.65 (d, *J* = 8.4 Hz, 2H), 7.56 (dd, *J* = 6.0, 1.8 Hz, 1H), 7.48-7.45 (m, 2H), 7.28 (d, *J* = 9.0 Hz, 2H), 5.77 (s, 2H); ^13^C NMR (151 MHz, DMSO-*d*_6_): *δ* 187.69, 176.12, 167.50, 161.70, 138.47, 137.04, 134.78, 132.83, 132.37, 131.75, 131.63, 131.63, 130.48, 130.01, 130.01, 129.37, 129.37, 129.05, 128.13, 125.14, 125.05, 115.37,115.37, 61.49. HRMS (ESI) m/z for C_24_H_17_O_3_N_2_Cl_2_ [M+H]^+^ calcd: 451.06107, found: 451.05978.

(*E*)-1-(4-((3-phenyl-1,2,4-oxadiazol-5-yl)methoxy)phenyl)-3-(p-tolyl)prop-2-en-1-one (**A10**). White powder; m.p. 169-170˚C; yield 80%; ^1^H NMR (400 MHz, DMSO-*d*_6_): *δ* 8.20 (d, *J* = 9.2 Hz, 2H), 8.03 (dd, *J* = 8.0, 1.6 Hz, 2H), 7.91 (d, *J* = 15.2 Hz, 1H), 7.78 (d, *J* = 8.0 Hz, 2H), 7.69 (d, *J* = 15.6 Hz, 1H), 7.63-7.57 (m, 3H), 7.29-7.26 (m, 4H), 5.77 (s, 2H), 2.36 (s, 3H); ^13^C NMR (101 MHz, DMSO-*d*_6_): *δ* 187.88, 175.93, 168.27, 161.47, 144.00, 141.04, 132.52, 132.31, 132.13, 131.40, 131.40, 130.00, 130.00, 129.85, 129.85, 129.36, 129.36, 127.57, 127.57, 126.20, 121.35, 115.29, 115.29, 61.48, 21.56. HRMS (ESI) m/z for C_25_H_21_O_3_N_2_ [M+H]^+^ calcd: 397.15467, found: 397.15359.

(*E*)-1-(4-((3-(4-fluorophenyl)-1,2,4-oxadiazol-5-yl)methoxy)phenyl)-3-(4-methoxyphenyl)prop-2-en-1-one (**A11**). White powder; m.p. 161-162˚C; yield 63%; ^1^H NMR (400 MHz, DMSO-*d*_6_): *δ* 8.19 (d, *J* = 8.8 Hz, 2H), 8.10-8.06 (m, 2H), 7.85 (d, *J* = 8.8 Hz, 2H), 7.82 (d, *J* = 15.6 Hz, 1H), 7.69 (d, *J* = 15.6 Hz, 1H), 7.43 (t, *J* = 8.8 Hz, 2H), 7.25 (d, *J* = 8.8 Hz, 2H), 7.01 (d, *J* = 8.8 Hz, 2H), 5.76 (s, 2H), 3.82 (s, 3H); ^13^C NMR (101 MHz, DMSO-*d*_6_): *δ* 187.77, 176.04, 167.48, 165.80 (C-F), 163.31 (C-F), 161.75, 161.34, 143.91, 132.30, 131.30, 131.30, 131.20, 131.20, 130.21, 130.12, 127.87, 122.77, 119.87, 117.14, 116.92, 115.24, 115.24, 114.85, 114.85, 61.45, 55.84. HRMS (ESI) m/z for C_25_H_20_O_4_N_2_F [M+H]^+^ calcd: 431.14016, found: 431.13901.

(*E*)-3-(4-methoxyphenyl)-1-(4-((3-(p-tolyl)-1,2,4-oxadiazol-5-yl)methoxy)phenyl)prop-2-en-1-one (**A12**). White powder; m.p. 167-168˚C; yield 78%; ^1^H NMR (400 MHz, DMSO-*d*_6_): *δ* 8.19 (d, *J* = 9.2 Hz, 2H), 7.91 (d, *J* = 8.0 Hz, 2H), 7.85 (d, *J* = 8.4 Hz, 2H), 7.83 (d, *J* = 15.6 Hz, 1H), 7.69 (d, *J* = 15.2 Hz, 1H), 7.39 (d, *J* = 8.0 Hz, 2H), 7.25 (d, *J* = 8.8 Hz, 2H), 7.02 (d, *J* = 8.8 Hz, 2H), 5.74 (s, 2H), 3.83 (s, 3H), 2.39 (s, 3H); ^13^C NMR (101 MHz, DMSO-*d*_6_): *δ* 187.14, 175.95, 167.87, 161.75, 160.78, 144.22, 141.83, 132.38, 131.31, 131.31, 131.21, 131.21, 130.39, 130.39, 128.01, 127.31, 127.31, 123.27, 119.86, 115.13, 115.13, 114.80, 114.80, 61.42, 55.99, 21.21. HRMS (ESI) m/z for C_26_H_23_O_4_N_2_ [M+H]^+^ calcd: 427.16523, found: 427.16388.

(*E*)-3-(4-methoxyphenyl)-1-(4-((3-phenyl-1,2,4-oxadiazol-5-yl)methoxy)phenyl)prop-2-en-1-one (**A13**). White powder; m.p. 149-150˚C; yield 66%; ^1^H NMR (400 MHz, DMSO-*d*_6_): *δ* 8.19 (d, *J* = 8.8 Hz, 2H), 8.03 (dd, *J* = 8.0, 1.6 Hz, 2H), 7.85 (d, *J* = 8.8 Hz, 2H), 7.82 (d, *J* = 15.6 Hz, 1H), 7.69 (d, *J* = 15.2 Hz, 1H), 7.63-7.56 (m, 3H), 7.26 (d, *J* = 8.8 Hz, 2H), 7.01 (d, *J* = 8.8 Hz, 2H), 5.76 (s, 2H), 3.82 (s, 3H); ^13^C NMR (101 MHz, DMSO-*d*_6_): *δ* 187.78, 175.94, 168.27, 161.75, 161.36, 143.92, 132.30, 132.29, 131.31, 131.31, 131.21, 131.21, 129.84, 129.84, 127.88, 127.57, 127.57, 126.20, 119.89, 115.25, 115.25, 114.86, 114.86, 61.48, 55.84. HRMS (ESI) m/z for C_25_H_21_O_4_N_2_ [M+H]^+^ calcd: 413.14858, found: 413.14847.

(*E*)-3-(3-methoxyphenyl)-1-(4-((3-phenyl-1,2,4-oxadiazol-5-yl)methoxy)phenyl)prop-2-en-1-one (**A14**). White powder; m.p. 137-138˚C; yield 79%; ^1^H NMR (400 MHz, DMSO-*d*_6_): *δ* 8.24 (d, *J* = 8.8 Hz, 2H), 8.04 (dd, *J* = 8.0, 1.6 Hz, 2H), 7.97 (d, *J* = 15.6 Hz, 1H), 7.71 (d, *J* = 15.2 Hz, 1H), 7.64-7.57 (m, 3H), 7.50 (s, 1H), 7.44 (d, *J* = 7.6 Hz, 1H), 7.37 (t, *J* = 8.0 Hz, 1H), 7.29 (d, *J* = 9.2 Hz, 2H), 7.03 (d, *J* = 8.0 Hz, 1H), 5.78 (s, 2H), 3.84 (s, 3H); ^13^C NMR (101 MHz, DMSO-*d*_6_): *δ* 187.92, 175.89, 168.27, 161.54, 160.12, 143.92, 136.64, 132.28, 132.02, 132.02, 131.50, 130.36, 129.82, 129.82, 127.57, 127.57, 126.21, 122.67, 122.13, 117.04, 115.29, 115.29, 113.82, 61.49, 55.76. HRMS (ESI) m/z for C_25_H_21_O_4_N_2_ [M+H]^+^ calcd: 413.14958, found: 413.14838.

(*E*)-1-(4-((3-(4-chlorophenyl)-1,2,4-oxadiazol-5-yl)methoxy)phenyl)-3-(2-fluorophenyl)prop-2-en-1-one (**A15**). White powder; m.p. 180-181˚C; yield 45%; ^1^H NMR (600 MHz, DMSO-*d*_6_): *δ* 8.20 (d, *J* = 9.0 Hz, 2H), 8.15-8.12 (m, 1H), 8.03 (d, *J* = 8.4 Hz, 2H), 8.00 (d, *J* =15.6 Hz, 1H), 7.83 (d, *J* = 15.6 Hz, 1H), 7.66 (d, *J* = 8.4 Hz, 2H), 7.54-7.50 (m, 1H), 7.33-7.32 (m, 2H), 7.28 (d, *J* = 9.0 Hz, 2H), 7.01 (d, *J* = 8.8 Hz, 2H), 5.77 (s, 2H); ^13^C NMR (151 MHz, DMSO-*d*_6_): *δ* 187.72, 176.14, 167.50, 162.20, 161.66, 160.53, 137.04, 135.01, 133.07, 131.79, 131.54, 131.54, 130.03, 130.03, 129.52, 129.39, 129.39, 125.43, 125.06, 124.50, 122.89 (C-F), 122.81 (C-F), 116.47, 115.40, 61.50. HRMS (ESI) m/z for C_24_H_17_O_3_N_2_ClF [M+H]^+^ calcd: 435.09062, found: 435.08942.

(E)-3-(2-methoxyphenyl)-1-(4-((3-phenyl-1,2,4-oxadiazol-5-yl)methoxy)phenyl)prop-2-en-1-one (**A16**). White powder; m.p. 124-125˚C; yield 83%; ^1^H NMR (400 MHz, DMSO-*d*_6_): *δ* 8.19 (d, *J* = 8.8 Hz, 2H), 8.07-8.03 (m, 3H), 7.98 (dd, *J* = 8.0, 1.6 Hz, 1H), 7.90 (d, *J* = 16.0 Hz, 1H), 7.63-7.57 (m, 3H), 7.48-7.43 (m, 1H), 7.27 (d, *J* = 9.2 Hz, 2H), 7.12 (d, *J* = 8.4 Hz, 1H), 7.04 (d, *J* = 7.6 Hz, 1H), 5.77 (s, 2H), 3.91 (s, 3H); ^13^C NMR (101 MHz, DMSO- *d*_6_): *δ* 188.04, 175.91, 168.27, 161.44, 158.67, 138.41, 132.66, 132.29, 132.18, 131.35, 131.35, 129.82, 129.82, 128.89, 127.57, 127.57, 126.21, 123.49, 122.17, 121.14, 115.30, 115.30, 112.24, 61.49, 56.18. HRMS (ESI) m/z for C_25_H_20_O_4_N_2_Na [M+Na]^+^ calcd: 435.13153, found: 435.13098.

(*E*)-3-(2-methoxyphenyl)-1-(4-((3-(p-tolyl)-1,2,4-oxadiazol-5-yl)methoxy)phenyl)prop-2-en-1-one (**A17**). Yellow powder; m.p. 133-134˚C; yield 81%; ^1^H NMR (400 MHz, DMSO-*d*_6_): *δ* 8.19 (d, *J* = 8.8 Hz, 2H), 8.05 (d, *J* = 15.6 Hz, 1H), 7.98 (d, *J* = 8.0 Hz, 1H), 7.94-7.88 (m, 3H), 7.45 (t, *J* = 7.6 Hz, 1H), 7.38 (d, *J* = 7.6 Hz, 2H), 7.27 (d, *J* = 8.4 Hz, 2H), 7.11 (d, *J* = 8.0 Hz, 1H), 7.04 (t, *J* = 7.6 Hz, 1H), 5.75 (s, 2H), 3.91 (s, 3H), 2.39 (s, 3H); ^13^C NMR (101 MHz, DMSO-*d*_6_): *δ* 188.04, 175.72, 168.23, 161.45, 158.67, 142.29, 138.40, 132.67, 132.17, 131.35, 131.35, 130.37, 130.37, 128.89, 127.51, 127.51, 123.48, 123.43, 122.17, 121.14, 115.29, 115.29, 112.25, 61.47, 56.18, 21.57. HRMS (ESI) m/z for C_26_H_22_O_4_N_2_Na [M+Na]^+^ calcd: 449.14718, found: 449.14661.

(*E*)-1-(4-((3-(4-fluorophenyl)-1,2,4-oxadiazol-5-yl)methoxy)phenyl)-3-(2-methoxyphenyl)prop-2-en-1-one (**A18**). White powder; m.p. 123-124˚C; yield 52%; ^1^H NMR (400 MHz, DMSO-*d*_6_): *δ* 8.19 (d, *J* = 8.8 Hz, 2H), 8.09 (dd, *J* = 8.8, 1.6 Hz, 2H), 8.06 (d, *J* = 16.0 Hz, 1H), 7.90 (d, *J* = 15.6 Hz, 1H), 7.43 (t, *J* = 8.8 Hz, 3H), 7.27 (d, *J* = 9.2 Hz, 2H), 7.12 (d, *J* = 8.4 Hz, 1H), 7.04 (t, *J* = 7.6 Hz, 1H), 5.77 (s, 2H), 3.91 (s, 3H); ^13^C NMR (101 MHz, DMSO-*d*_6_): *δ* 188.03, 176.00, 167.48, 165.79 (C-F), 163.31 (C-F), 161.42, 158.67, 138.41, 132.64, 132.19, 131.34, 131.34, 130.20, 130.11, 128.88, 123.49, 122.80, 122.15, 121.13, 117.11, 116.89, 115.29, 115.29, 112.22, 61.46, 56.16. HRMS (ESI) m/z for C_25_H_19_O_4_N_2_FNa [M+Na]^+^ calcd: 453.12211, found: 453.12125.

(*E*)-1-(4-((3-phenyl-1,2,4-oxadiazol-5-yl)methoxy)phenyl)-3-(2-(trifluoromethyl)phenyl)prop-2-en-1-one (**A19**). White powder; m.p. 158-159˚C; yield 51%; ^1^H NMR (400 MHz, DMSO-*d*_6_): *δ* 8.34 (d, *J* = 7.6 Hz, 1H), 8.25 (d, *J* = 9.2 Hz, 2H), 8.08-7.95 (m, 4H), 7.85-7.78 (m, 2H), 7.69-7.57 (m, 4H), 7.30 (d, *J* = 9.2 Hz, 2H), 5.79 (s, 2H); ^13^C NMR (101 MHz, DMSO-*d*_6_): *δ* 187.55, 175.85, 168.27, 161.83, 137.75, 133.39, 132.28, 131.71, 131.71, 131.56, 130.86, 129.81, 129.81, 129.25, 127.93 (d, *J* = 19.49 Hz), 127.56, 127.56, 127.52, 126.64, 126.59, 126.20, 126.02 (q, *J* = 274.02 Hz), 115.40, 115.40, 61.52. HRMS (ESI) m/z for C_25_H_17_O_3_N_2_F_3_Na [M+Na]^+^ calcd: 473.10835, found: 473.10760.

(*E*)-3-(3-methoxyphenyl)-1-(4-((3-(p-tolyl)-1,2,4-oxadiazol-5-yl)methoxy)phenyl)prop-2-en-1-one (**A20**). White powder; m.p. 157-158˚C; yield 72%; ^1^H NMR (600 MHz, DMSO-*d*_6_): *δ* 8.22 (d, *J* = 8.4 Hz, 2H), 7.96 (d, *J* = 15.0 Hz, 1H), 7.92 (d, *J* = 8.4 Hz, 2H), 7.70 (d, *J* = 15.6 Hz, 1H), 7.49 (s, 1H), 7.43 (d, *J* = 7.8 Hz, 1H), 7.38 (d, *J* = 7.8 Hz, 2H), 7.36 (d, *J* = 7.8 Hz, 1H), 7.27 (d, *J* = 9.0 Hz, 2H), 7.02 (dd, *J* = 7.8, 1.8 Hz, 1H), 5.75 (s, 2H), 3.83 (s, 3H), 2.39 (s,3H); ^13^C NMR (151 MHz, DMSO-*d*_6_): *δ* 187.92, 175.71, 168.23, 161.56, 160.12, 143.92, 142.30, 136.64, 132.01, 131.50, 131.50, 130.37, 130.37, 130.37, 127.51, 127.51, 123.43, 122.67, 122.14, 117.05, 115.29, 115.29, 113.81, 61.48, 55.77, 21.56. HRMS (ESI) m/z for C_26_H_22_O_4_N_2_Na [M+Na]^+^ calcd: 449.14718, found: 449.14633.

(*E*)-3-(4-bromophenyl)-1-(4-((3-phenyl-1,2,4-oxadiazol-5-yl)methoxy)phenyl)prop-2-en-1-one (**A21**). White powder; m.p. 196-197˚C; yield 66%; ^1^H NMR (600 MHz, DMSO-*d*_6_): *δ* 8.22 (d, *J* = 9.0 Hz, 2H), 8.03 (dd, *J* = 6.6, 1.8 Hz, 2H), 8.01 (d, *J* = 15.6 Hz, 1H), 7.86 (d, *J* = 8.4 Hz, 2H), 7.69 (d, *J* = 15.6 Hz, 1H), 7.66 (d, *J* = 8.4 Hz, 2H), 7.63-7.57 (m, 3H), 7.27 (d, *J* = 8.4 Hz, 2H), 5.77 (s, 2H); ^13^C NMR (151 MHz, DMSO-*d*_6_): *δ* 187.80, 175.89, 168.27, 161.61, 142.53, 134.56, 132.32, 132.32, 131.92, 131.53, 131.53, 131.23, 131.23, 129.84, 129.84, 127.57, 127.57, 127.57, 126.20, 124.32, 123.22, 115.33, 115.33, 61.50. HRMS (ESI) m/z for C_24_H_18_O_3_N_2_Br [M+H]^+^ calcd: 461.04953, found: 461.04834.

*(E)-1-(2,4-dichlorophenyl)-3-(4-((3-phenyl-1,2,4-oxadiazol-5-yl)methoxy)phenyl)prop-2-en-1-one* (**B1**). Faint yellow powder; m.p. 126-127˚C; yield 56%; ^1^H NMR (400 MHz, DMSO-*d_6_*): *δ* 8.33-8.27 (m, 3H), 8.12-7.98 (m, 4H), 7.79 (d, *J* = 1.6 Hz, 1H), 7.67-7.59 (m, 4H), 7.33 (d, *J* = 8.8 Hz, 2H), 5.82 (s, 2H); ^13^C NMR (101 MHz, DMSO-*d_6_*): *δ* 187.54, 175.87, 168.27, 161.79, 137.19, 135.98, 135.57, 132.31, 131.92, 131.67, 131.67, 131.65, 130.30, 129.95, 129.84, 129.84, 128.39, 127.57, 127.57, 126.20, 125.72, 115.39, 115.39, 61.51. HRMS (ESI) m/z for C_24_H_17_O_3_N_2_Cl_2_ [M+H]^+^ calcd: 451.06107, found: 451.05972.

(*E*)-1-(2,4-dichlorophenyl)-3-(4-((3-(p-tolyl)-1,2,4-oxadiazol-5-yl)methoxy)phenyl)prop-2-en-1-one (**B2**). Faint yellow powder; m.p. 156-157˚C; yield 72%; ^1^H NMR (400 MHz, DMSO-*d_6_*): *δ* 8.26 (d, *J* = 8.8 Hz, 1H), 8.22 (d, *J* = 8.8 Hz, 2H), 8.04 (d, *J* = 15.6 Hz, 1H), 7.94 (d, *J* = 16.4 Hz, 1H), 7.90 (d, *J* = 8.4 Hz, 2H), 7.73 (d, *J* = 2.0 Hz, 1H), 7.54 (dd, *J* = 8.8, 2.0 Hz, 1H), 7.38 (d, *J* = 8.0 Hz, 2H), 7.27 (d, *J* = 8.8 Hz, 2H), 5.75 (s, 2H), 2.38 (s, 3H); ^13^C NMR (101 MHz, DMSO-*d_6_*): *δ* 187.54, 175.68, 168.23, 161.80, 142.31, 137.19, 135.98, 135.57, 131.92, 131.67, 131.67, 131.64, 130.38, 130.38, 130.30, 129.96, 128.39, 127.51, 127.51, 125.72, 123.42, 115.38, 115.38, 61.50, 21.57. HRMS (ESI) m/z for C_25_H_19_O_3_N_2_Cl_2_ [M+H]^+^ calcd: 465.07672, found: 465.07544.

(*E*)-3-(4-((3-(4-chlorophenyl)-1,2,4-oxadiazol-5-yl)methoxy)phenyl)-1-(2,4-dichlorophenyl)prop-2-en-1-one (**B3**). White powder; m.p. 187-188˚C; yield 41%; ^1^H NMR (400 MHz, CDCl_3_): *δ* 8.14-8.05 (m, 5H), 7.70 (d, *J* = 8.4 Hz, 1H), 7.51-7.47 (m, 4H), 7.32 (dd, *J* = 8.8, 2.0 Hz, 1H), 7.15 (d, *J* = 8.8 Hz, 2H), 5.47 (s, 2H); ^13^C NMR (101 MHz, CDCl_3_): *δ* 188.30, 174.26, 167.93, 161.14, 139.09, 137.81, 136.36, 136.07, 132.18, 131.88, 131.10, 131.10, 130.18, 129.34, 129.34, 128.88, 128.88, 128.50, 127.57, 124.66, 124.57, 114.71, 114.71, 60.90. HRMS (ESI) m/z for C_24_H_16_O_3_N_2_Cl_2_ [M+H]^+^ calcd: 485.02210, found: 485.02121.

(*E*)-1-(2,4-dichlorophenyl)-3-(4-((3-(4-fluorophenyl)-1,2,4-oxadiazol-5-yl)methoxy)phenyl)prop-2-en-1-one (**B4**). White powder; m.p. 152-153˚C; yield 43%; ^1^H NMR (400 MHz, DMSO-*d_6_*): *δ* 8.30 (d, *J* = 8.4 Hz, 1H), 8.27 (d, *J* = 8.8 Hz, 2H), 8.14-8.11 (m, 2H), 8.09 (d, *J* = 15.2 Hz, 1H), 7.99 (d, *J* = 15.6 Hz, 1H), 7.78 (d, *J* = 2.0 Hz, 1H), 7.59 (dd, *J* = 8.8, 2.0 Hz, 1H), 7.47 (t, *J* = 8.8 Hz, 2H), 7.32 (d, *J* = 8.8 Hz, 2H), 5.81 (s, 2H); ^13^C NMR (101 MHz, DMSO-*d_6_*): *δ* 187.36, 175.81, 167.32, 165.63, 163.15, 161.60, 137.02, 135.81, 135.40, 131.75, 131.50, 131.50, 130.12, 130.04, 129.95, 129.78, 128.21, 125.52, 122.62, 116.97, 116.75, 115.21, 115.21, 61.33. HRMS (ESI) m/z for C_24_H_6_O_3_N_2_Cl_2_F [M+H]^+^ calcd: 469.05165, found: 469.05026.

(*E*)-1-phenyl-3-(4-((3-phenyl-1,2,4-oxadiazol-5-yl)methoxy)phenyl)prop-2-en-1-one (**B5**). White powder; m.p. 177-178˚C; yield 49%; ^1^H NMR (400 MHz, DMSO-*d_6_*): *δ* 8.14 (d, *J* = 7.2 Hz, 2H), 8.04 (dd, *J* = 7.6, 1.6 Hz, 2H), 7.91 (d, *J* = 8.8 Hz, 2H), 7.86 (d, *J* = 15.6 Hz, 1H), 7.74 (d, *J* = 15.6 Hz, 1H),7.69 (t, *J* = 7.2 Hz, 1H), 7.62-7.55 (m, 5H), 7.20 (d, *J* = 8.8 Hz, 2H), 5.71 (s, 2H); ^13^C NMR (101 MHz, DMSO-*d_6_*): *δ* 189.53, 176.07, 168.25, 159.67, 144.07, 138.21, 133.49, 132.31, 131.34, 131.34, 129.85, 129.85, 129.24,129.24, 129.07, 128.93, 128.93, 127.57, 127.57, 126.22, 120.83, 115.71, 115.71, 61.41. HRMS (ESI) m/z for C_24_H_19_O_3_N_2_ [M+H]^+^ calcd: 383.13902, found: 383.13788.

(*E*)-1-phenyl-3-(4-((3-(p-tolyl)-1,2,4-oxadiazol-5-yl)methoxy)phenyl)prop-2-en-1-one (**B6**). White powder; m.p. 194-195˚C; yield 35%; ^1^H NMR (400 MHz, DMSO-*d*_6_): *δ* 8.14 (d, *J* = 7.2 Hz, 2H), 7.92 (d, *J* = 8.4 Hz, 4H), 7.86 (d, *J* = 15.6 Hz, 1H), 7.73 (d, *J* = 15.2 Hz, 1H), 7.67 (t, *J* = 7.2 Hz, 1H), 7.57 (t, *J* = 8.0 Hz, 2H), 7.39 (d, *J* = 8.0 Hz, 2H), 7.19 (d, *J* = 8.8 Hz, 2H), 5.69 (s, 2H), 2.39 (s, 3H); ^13^C NMR (101 MHz, CDCl_3_): *δ* 190.43, 174.11, 168.67, 159.33, 144.12, 141.99, 138.33, 132.75, 130.33, 130.33, 129.69, 129.69, 129.24, 128.64, 128.64, 128.48, 128.48, 127.49, 127.49, 123.29, 120.71, 115.27, 115.27, 61.03, 21.65. HRMS (ESI) m/z for C_25_H_21_O_3_N_2_ [M+H]^+^ calcd: 397.15467, found: 397.15344.

(*E*)-3-(4-((3-(4-chlorophenyl)-1,2,4-oxadiazol-5-yl)methoxy)phenyl)-1-phenylprop-2-en-1-one (**B7**). White powder; m.p. 200-201˚C; yield 47%; ^1^H NMR (400 MHz, DMSO-*d_6_*): *δ* 8.14 (d, *J* = 7.2 Hz, 2H), 8.04 (d, *J* = 8.4 Hz, 2H), 7.92 (d, *J* = 8.4 Hz, 2H), 7.86 (d, *J* = 15.6 Hz, 1H), 7.73 (d, *J* = 15.6 Hz, 1H), 7.66 (d, *J* = 8.4 Hz, 3H), 7.57 (t, *J* = 7.6 Hz, 2H), 7.20 (d, *J* = 8.4 Hz, 2H), 5.71 (s, 2H); ^13^C NMR (151 MHz, DMSO-*d_6_*): *δ* 189.53, 176.31, 167.48, 159.64, 144.06, 138.20, 137.03, 133.49, 131.33, 131.33, 130.02, 130.02, 129.38, 129.38, 129.23, 129.23, 129.08, 128.92, 128.92, 125.08, 120.83, 115.70, 115.70, 61.39. HRMS (ESI) m/z for C_24_H_18_O_3_N_2_Cl [M+H]^+^ calcd: 417.10005, found: 417.09872.

(*E*)-3-(4-((3-(4-fluorophenyl)-1,2,4-oxadiazol-5-yl)methoxy)phenyl)-1-phenylprop-2-en-1-one (**B8**). White powder; m.p. 187-188˚C; yield 72%; ^1^H NMR (400 MHz, CDCl_3_): 8.15-8.11 (m, 2H), 8.03 (d, *J* = 7.6 Hz, 2H), 7.81 (d, *J* = 15.6 Hz, 1H), 7.67 (d, *J* = 8.8 Hz, 2H), 7.60 (d, *J* = 7.6 Hz, 1H), 7.53 (t, *J* = 7.6 Hz, 2H), 7.47 (d, *J* = 15.6 Hz, 1H), 7.21 (t, *J* = 8.8 Hz, 2H), 7.10 (d, *J* = 8.8 Hz, 2H), 5.42 (s, 2H); ^13^C NMR (101 MHz, DMSO-*d_6_*): *δ* 190.50, 174.42, 167.86, 163.50, 159.26, 144.05, 138.30, 132.77, 130.34, 130.34, 128.65, 128.48, 129.82, 129.73, 129.26, 128.65, 128.48, 120.76, 118.21, 116.33, 116.11, 115.25, 115.25, 60.97. HRMS (ESI) m/z for C_24_H_18_O_3_N_2_F [M+H]^+^ calcd: 401.12960, found: 401.12848.

(*E*)-1-(2-chlorophenyl)-3-(4-((3-(4-chlorophenyl)-1,2,4-oxadiazol-5-yl)methoxy)phenyl)prop-2-en-1-one (**B9**). White powder; m.p. 141-142˚C; yield 68%; ^1^H NMR (400 MHz, DMSO-*d_6_*): *δ* 8.02 (d, *J* = 8.4 Hz, 2H), 7.78 (d, *J* = 8.8 Hz, 2H), 7.65 (d, *J* = 8.4 Hz, 2H), 7.60-7.48 (m, 4H), 7.38 (d, *J* = 16.0 Hz, 1H), 7.20-7.16 (m, 3H), 5.70 (s, 2H); ^13^C NMR (101 MHz, DMSO-*d_6_*): *δ* 193.60, 176.23, 167.47, 159.95, 146.33, 139.31, 137.03, 132.16, 131.39, 131.39, 130.53, 130.34, 130.00, 130.00, 129.67, 129.36, 129.36, 128.41, 127.84, 125.16, 125.06, 115.79, 115.79, 61.38. HRMS (ESI) m/z for C_24_H_17_O_3_N_2_Cl_2_ [M+H]^+^ calcd: 451.06107, found: 451.05972.

(*E*)-3-(4-((3-phenyl-1,2,4-oxadiazol-5-yl)methoxy)phenyl)-1-(p-tolyl)prop-2-en-1-one (**B10**). White powder; m.p. 193-194˚C; yield 54%; ^1^H NMR (600 MHz, DMSO-*d_6_*): *δ* 8.05 (d, *J* = 8.4 Hz, 2H), 8.02 (d, *J* = 6.6 Hz, 2H), 7.89 (d, *J* = 9.0 Hz, 2H), 7.83 (d, *J* = 15.6 Hz, 1H), 7.71 (d, *J* = 15.0 Hz, 1H), 7.61-7.56 (m, 3H), 7.36 (d, *J* = 8.4 Hz, 2H), 7.18 (d, *J* = 9.0 Hz, 2H), 5.70 (s, 2H), 2.39 (s, 3H); ^13^C NMR (151 MHz, DMSO-*d_6_*): *δ* 188.95, 176.05, 168.25, 159.59, 143.88, 143.66, 135.69, 132.28, 131.25, 129.83, 129.83, 129.83, 129.79, 129.79, 129.14, 129.07, 129.07, 127.56, 127.57, 126.22, 120.83, 115.68, 115.68, 61.40, 21.65. HRMS (ESI) m/z for C_25_H_21_O_3_N_2_ [M+H]^+^ calcd: 397.15467, found: 397.15344.

(*E*)-3-(4-((3-(4-fluorophenyl)-1,2,4-oxadiazol-5-yl)methoxy)phenyl)-1-(4-methoxyphenyl)prop-2-en-1-one (**B11**). White powder; m.p. 188-189˚C; yield 49%; ^1^H NMR (600 MHz, DMSO-*d_6_*): *δ* 8.16 (d, *J* = 9.0 Hz, 2H), 8.08 (dd, *J* = 9.0, 5.4 Hz, 2H), 7.89 (d, *J* = 8.4 Hz, 2H), 7.85 (d, *J* = 15.6 Hz, 1H), 7.69 (d, *J* = 15.6 Hz, 1H), 7.42 (t, *J* = 8.4 Hz, 2H), 7.19 (d, *J* = 8.4 Hz, 2H), 7.08 (d, *J* = 9.0 Hz, 1H), 5.70 (s, 2H), 3.34 (s, 3H); ^13^C NMR (151 MHz, DMSO-*d_6_*): *δ* 187.73, 176.18, 167.47, 165.38, 163.72, 163.60, 159.48, 143.15, 131.30, 131.30, 131.17, 131.17, 131.07, 130.19, 130.12, 129.24, 122.80, 120.79, 117.09, 116.95, 115.66, 114.44, 114.44, 61.38, 56.02. HRMS (ESI) m/z for C_25_H_20_O_4_N_2_F [M+H]^+^ calcd: 431.14016, found: 431.13910.

(*E*)-1-(4-methoxyphenyl)-3-(4-((3-(p-tolyl)-1,2,4-oxadiazol-5-yl)methoxy)phenyl)prop-2-en-1-one (**B12**). White powder; m.p. 184-185˚C; yield 66%; ^1^H NMR (600 MHz, DMSO-*d_6_*): *δ* 8.16 (d, *J* = 9.0 Hz, 2H), 7.91 (d, *J* = 8.4 Hz, 2H), 7.89 (d, *J* = 9.0 Hz, 2H), 7.85 (d, *J* = 15.6 Hz, 1H), 7.69 (d, *J* = 15.6 Hz, 1H), 7.38 (d, *J* = 7.8 Hz, 2H), 7.19 (d, *J* = 9.0 Hz, 2H), 7.08 (d, *J* = 8.4 Hz, 2H), 5.68 (s, 2H), 3.87 (s, 3H), 2.39 (s, 3H); ^13^C NMR (151 MHz, DMSO-*d_6_*): *δ* 187.73, 175.88, 168.22, 163.60, 159.50, 143.16, 142.28, 131.31, 131.31, 131.17, 131.17, 131.07, 130.37, 130.37, 129.22, 127.51, 127.51, 123.45, 120.78, 115.65, 115.65, 114.45, 114.45, 61.39, 56.03, 21.57. HRMS (ESI) m/z for C_26_H_23_O_4_N_2_ [M+H]^+^ calcd: 427.16523, found: 427.16403.

(*E*)-3-(4-((3-(4-chlorophenyl)-1,2,4-oxadiazol-5-yl)methoxy)phenyl)-1-(4-methoxyphenyl)prop-2-en-1-one (**B13**). White powder; m.p. 201-202˚C; yield 39%; ^1^H NMR (600 MHz, DMSO-*d_6_*): *δ* 8.15 (d, *J* = 9.0 Hz, 2H), 8.03 (d, *J* = 8.4 Hz, 2H), 7.89 (d, *J* = 8.4 Hz, 2H), 7.84 (d, *J* = 15.6 Hz, 1H), 7.69 (d, *J* = 15.6 Hz, 1H), 7.65 (d, *J* = 8.4 Hz, 2H), 7.19 (d, *J* = 8.4 Hz, 2H), 7.08 (d, *J* = 9.0 Hz, 2H), 5.70 (s, 2H), 3.87 (s, 3H); ^13^C NMR (151 MHz, DMSO-*d_6_*): *δ* 187.73, 176.34, 167.48, 163.61, 159.47, 143.15, 137.03, 131.31, 131.31, 131.18, 131.18, 131.06, 130.03, 130.03, 129.38, 129.38, 129.25, 125.09, 120.81, 115.66, 115.66, 114.46, 114.46, 61.39, 56.04. HRMS (ESI) m/z for C_25_H_20_O_4_N_2_Cl [M+H]^+^ calcd: 447.11061, found: 447.10925.

**2. ^1^H NMR, ^13^C NMR, and HRMS spectra of target compounds A1‒A21 and B1‒B13**

^1^H NMR of compound **A1**

^13^C NMR of compound **A1**

HRMS of compound **A1**

^1^H NMR of compound **A2**

^13^C NMR of compound **A2**

HRMS of compound **A2**

^1^H NMR of compound **A3**

^13^C NMR of compound **A3**

HRMS of compound **A3**

^1^H NMR of compound **A4**

^13^C NMR of compound **A4**

HRMS of compound **A4**

^1^H NMR of compound **A5**

^13^C NMR of compound **A5**

HRMS of compound **A5**

^1^H NMR of compound **A6**

^13^C NMR of compound **A6**

HRMS of compound **A6**

^1^H NMR of compound **A7**

^13^C NMR of compound **A7**

HRMS of compound **A7**

^1^H NMR of compound **A8**

^13^C NMR of compound **A8**

HRMS of compound **A8**

^1^H NMR of compound **A9**

^13^C NMR of compound **A9**

HRMS of compound **A9**

^1^H NMR of compound **A10**

^13^C NMR of compound **A10**

HRMS of compound **A10**

^1^H NMR of compound **A11**

^13^C NMR of compound **A11**

HRMS of compound **A11**

^1^H NMR of compound **A12**

^13^C NMR of compound **A12**

HRMS of compound **A12**

^1^H NMR of compound **A13**

^13^C NMR of compound **A13**

HRMS of compound **A13**

^1^H NMR of compound **A14**

^13^C NMR of compound **A14**

HRMS of compound **A14**

^1^H NMR of compound **A15**

^13^C NMR of compound **A15**

HRMS of compound **A15**

^1^H NMR of compound **A16**

^13^C NMR of compound **A16**

HRMS of compound **A16**

^1^H NMR of compound **A17**

^13^C NMR of compound **A17**

HRMS of compound **A17**

^1^H NMR of compound **A18**

^13^C NMR of compound **A18**

HRMS of compound **A18**

^1^H NMR of compound **A19**

^13^C NMR of compound **A19**

HRMS of compound **A19**

^1^H NMR of compound **A20**

^13^C NMR of compound **A20**

HRMS of compound **A20**

^1^H NMR of compound **A21**

^13^C NMR of compound **A21**

HRMS of compound **A21**

^1^H NMR of compound **B1**

^13^C NMR of compound **B1**

HRMS of compound **B1**

^1^H NMR of compound **B2**

^13^C NMR of compound **B2**

HRMS of compound **B2**

^1^H NMR of compound **B3**

^13^C NMR of compound **B3**

HRMS of compound **B3**

^1^H NMR of compound **B4**

^13^C NMR of compound **B4**

HRMS of compound **B4**

^1^H NMR of compound **B5**

^13^C NMR of compound **B5**

HRMS of compound **B5**

^1^H NMR of compound **B6**

^13^C NMR of compound **B6**

HRMS of compound **B6**

^1^H NMR of compound **B7**

^13^C NMR of compound **B7**

HRMS of compound **B7**

^1^H NMR of compound **B8**

^13^C NMR of compound **B8**

HRMS of compound **B8**

^1^H NMR of compound **B9**

^13^C NMR of compound **B9**

HRMS of compound **B9**

^1^H NMR of compound **B10**

^13^C NMR of compound **B10**

HRMS of compound **B10**

^1^H NMR of compound **B11**

^13^C NMR of compound **B11**

HRMS of compound **B11**

^1^H NMR of compound **B12**

^13^C NMR of compound **B12**

HRMS of compound **B12**

^1^H NMR of compound **B13**

^13^C NMR of compound **B13**

HRMS of compound **B13**
